# Advances in Natural-Product-Based Fluorescent Agents and Synthetic Analogues for Analytical and Biomedical Applications

**DOI:** 10.3390/bioengineering11121292

**Published:** 2024-12-19

**Authors:** Soniya Joshi, Alexis Moody, Padamlal Budthapa, Anita Gurung, Rachana Gautam, Prabha Sanjel, Aakash Gupta, Surya P. Aryal, Niranjan Parajuli, Narayan Bhattarai

**Affiliations:** 1 Central Department of Chemistry, Tribhuvan University, Kathmandu 44618, Nepal; soniyajoshi157@gmail.com (S.J.); padambudthapa8@gmail.com (P.B.); anitagurung9855@gmail.com (A.G.); rachanag759@gmail.com (R.G.); prabhasanjel01@gmail.com (P.S.); 2 Department of Chemical, Biological, and Bioengineering, North Carolina A&T State University, Greensboro, NC 27411, USA; amoody@aggies.ncat.edu; 3 Department of Biomedical Engineering, University of Wisconsin-Milwaukee, Milwaukee, WI 53211, USA; gupta_aakash_007@yahoo.com; 4 Department of Chemistry, University of Kentucky, Lexington, KY 40506, USA; aryalpsurya@gmail.com

**Keywords:** natural fluorescent, fluorescence properties, drug delivery, biomarkers, cancer

## Abstract

Fluorescence is a remarkable property exhibited by many chemical compounds and biomolecules. Fluorescence has revolutionized analytical and biomedical sciences due to its wide-ranging applications in analytical and diagnostic tools of biological and environmental importance. Fluorescent molecules are frequently employed in drug delivery, optical sensing, cellular imaging, and biomarker discovery. Cancer is a global challenge and fluorescence agents can function as diagnostic as well as monitoring tools, both during early tumor progression and treatment monitoring. Many fluorescent compounds can be found in their natural form, but recent developments in synthetic chemistry and molecular biology have allowed us to synthesize and tune fluorescent molecules that would not otherwise exist in nature. Naturally derived fluorescent compounds are generally more biocompatible and environmentally friendly. They can also be modified in cost-effective and target-specific ways with the help of synthetic tools. Understanding their unique chemical structures and photophysical properties is key to harnessing their full potential in biomedical and analytical research. As drug discovery efforts require the rigorous characterization of pharmacokinetics and pharmacodynamics, fluorescence-based detection accelerates the understanding of drug interactions via in vitro and in vivo assays. Herein, we provide a review of natural products and synthetic analogs that exhibit fluorescence properties and can be used as probes, detailing their photophysical properties. We have also provided some insights into the relationships between chemical structures and fluorescent properties. Finally, we have discussed the applications of fluorescent compounds in biomedical science, mainly in the study of tumor and cancer cells and analytical research, highlighting their pivotal role in advancing drug delivery, biomarkers, cell imaging, biosensing technologies, and as targeting ligands in the diagnosis of tumors.

## 1. Introduction

The word “fluorescence” was first coined in 1852 when George Gabriel Stokes published a paper on the change in the refrangibility of light, demonstrating how quinine reacts with ultraviolet light to produce a blue color [1]. It is typically short-lived (a few nanoseconds) and does not involve intersystem crossing, which is observed when an excited molecule or atom relaxes to a lower energy state by emitting a photon without reversing its spin multiplicity [2]. Fluorescence emission is intimately linked to the processes of excitation and emission: a photon of a particular wavelength is absorbed by a molecule, which, in turn, excites the molecule to a higher energy state. Subsequently, longer wavelength photons are re-emitted, which is an essential feature of most natural products with inherent optical characteristics [3]. The photon energy emitted by the molecule is always less than the absorbed photon due to the non-radioactive decay (internal conversion), so the efficiency or quantum yield of the molecule is always less than one. Fluorescence is strongest in the molecule with a quantum yield of one. Nevertheless, the molecules having a quantum yield of φ = 0.1 are also quite fluorescent.

Naturally derived fluorescent compounds have attracted increasing attention due to their widespread applications in surgical molecular navigation, live cell imaging [4], biosensing [5], chemical sensing, and protein tagging [6]. Their affinity for biological macromolecules and fluorescence characteristics justify their use in these different applications [7]. These affordable and easy-to-use probes can be employed in bioimaging to track and monitor in vivo physiological responses in disease situations, identify particular biomolecules, and, specifically, understand cellular and tissue morphology without invasive methods [8]. Important groups of fluorescence probes are built on the fundamental structures of secondary metabolites of living organisms, such as coumarins, acridines, quinolones, azulenes, and anthraquinones [9]. The structure of a fluorescent compound plays a crucial role in its optical properties. For instance, long alkyl chains and halogen atoms produce a high-contrast, vibrant mechanical response that may be applied for different applications, including color painting [10].

Natural products with fluorescent properties can be utilized in fluorescent probes, which have significantly improved the study of cell biology and chemical biology, leading to advancements in biomedical research [11]. While synthetic modifications and variations in functional groups can enhance the properties of fluorescent compounds, not all natural products are suitable for conversion into biochemically active probes. Epicocconone is a rare but great illustration of how natural fluorescent compounds are not easily converted into biochemical probes, although many fluorescent dyes are generated from natural substrates because of their low quantum yield and photostability [12]. In contrast, some synthetic analogs, such as pyrene, carbostyril 124, and 1,2,4–trioxolane are frequently used as fluorescent agents, because of their higher photophysical qualities and have demonstrated promising results in biomedical applications [13].

Cancer cells express numerous surface proteins that are generally absent in normal healthy cells. These proteins enable cancer cells to proliferate through alternative metabolic pathways and metastasize from one tissue to another. Many such targets are used for therapeutic intervention but can also serve as diagnostic markers, making them amenable to antibodies, small molecules, and aptamer-based fluorescence targeting and imaging approaches. Fluorescent probes can be valuable in three primary aspects of cancer biology: early cancer diagnostics using cancer biomarkers, the monitoring of therapeutic agents to assess their on-target and off-target effects, and evaluating bodily responses to therapeutics, including the activation of physiological pathways and immune cell activity [14]. Yao et al. synthesized and analyzed the pharmacological efficacy and subcellular distribution of some fluorescent 23-hydroxybetulinic acid (HBA) derivatives coupled with coumarin dyes [15]. While fluorescence has been explored in diverse fields, the application of fluorescence fingerprinting in cancer diagnostics is promising [16]. By using this technology, researchers can develop fluorescence libraries to identify cancer-specific biomarkers, enabling more precise and noninvasive diagnostic techniques. For example, fluorescent probes have been used to monitor biological samples in real-time and detect metal ions and bioactive compounds [17]. Additionally, curcumin, a compound with anticancer effects serves as a fluorescent probe for monitoring noisome migration within cells, which is advantageous for researching its fluorescence-switching abilities [18]. The high surgical recurrence incidence of pancreatic ductal adenocarcinoma, a lethal, aggressive tumor, can be observed and analyzed using a cellular mesenchymal-epithelial transformation factor-targeted, near-infrared fluorescence probe [16].

In addition, natural-product-derived fluorescent products have shown analytical utility; for example, Xu et al. prepared widely diffuse and water-soluble fluorescent nanodots from potatoes with a quantum yield of (0.15) and intense blue fluorescence that can be used as a novel sensing probe for the accurate and focused detection of Fe^3+^ ions in lake water [17]. In this review, we discuss the recent developments of small fluorescent probes based on natural products and their synthetic analogs that have significant applications in the biological, biochemical, and biomedical fields.

## 2. Natural Fluorescent Compounds

Since ancient times, scientists have been captivated by the usefulness of naturally occurring tiny molecules generated from plants, animals, and prokaryotes. These organisms have inherent optical properties and fluorescence emissions and are useful for tracking medications in biological systems and providing spatial information [3].

Tryptophan (Trp), tyrosine (Tyr), and phenylalanine (Phe) are the inherent aromatic amino acids that primarily cause the fluorescence signals from folded proteins. The signals from these aromatic amino acids have been consistently investigated to assess protein behavior, such as folding and unfolding; dynamics; pH and temperature dependence; cellular functions; sensing in an aqueous environment; etc. Despite this, their low molar extinction coefficient, low quantum yield, and sensitivity to the environment limit the utilization of these natural fluorophores in in vivo applications [18,19,20]. The first naturally occurring fluorescent protein produced by living organisms was green fluorescent protein (GFP), which acts as a light-emitting agent and is widely used in cell imaging, as well as in vitro and in vivo assays. It was first discovered in the jellyfish *Aequorea victoria* and, thereafter, was found in other organisms, including the sea pansy *Renilla reniformis* and many coelenterates [21,22]. When expressed in prokaryotic *(Escherichia coli*) or eukaryotic (*Caenorhabditis elegans*) cells, the gene for the protein results in a fluorescent product, which glows without the need for external substrates or cofactors; thus, its expression can be utilized to track the expression and localization of proteins in living organisms [23]. In addition, it is frequently employed as a marker of gene expression due to its stability and ability to generate chromophores after autocatalytic cyclization [24]. Modified GFP probes, such as GCaMPs, have been widely used for monitoring the real-time activity of Ca^2+^ ions in both cellular and subcellular settings [25]. The ability to visualize and investigate the processes and steps of metastasis is complicated and mysterious, and the key to breaking the chain of cancer can be facilitated by using GFPs as a tagging agent [26]. Proteins can be fluorescently tagged with GFPs at their N- or C-termini for visualization. In addition to GFPs, small molecule fluorescent probes are utilized to identify and quantify reactive oxygen and nitrogen species in redox biology and biochemistry. They can function as sensors for the direct assessment of biological events, including pH changes and the concentrations of different cellular ions [27]. Although the proteins that can be genetically altered have brought a revolution in in vitro and in vivo studies, they typically have lower luminescence and photostability, compared to synthetic fluorescent compounds. To improve these properties, photostabilizers that covalently connect within the fluorescent proteins have been rediscovered, enhancing the photophysical characteristics of fluorescent proteins [28].

**Figure 1:**
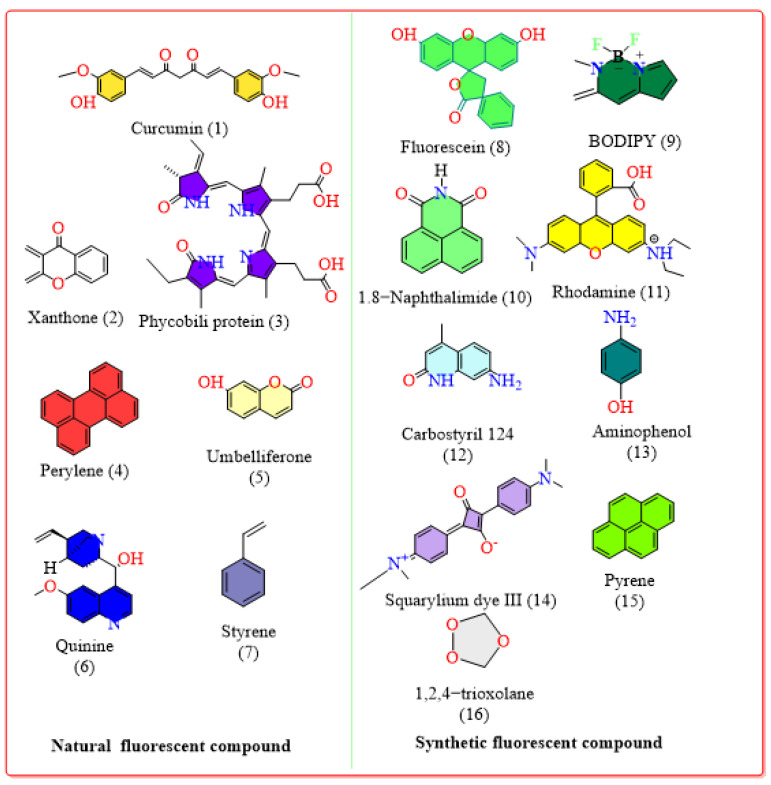
Chemical structures of selected natural products and synthetic fluorescent compounds. Color represents the fluorescence of the respective structures.

Moreover, curcumin, coumarin, quinine, *R*-phycoerythrin, and perylene are some of the fluorescent compounds isolated as natural products and used for various purposes. Curcumin, found in turmeric, can inhibit the mutagenesis of DNA and can also be used to visualize functionalized graphene nanosheets, which, in turn, can be used in various applications, including disease diagnosis, bioimaging, drug delivery, and so on [29,30,31]. Coumarin can be isolated from *Cortex fraxinus* and can be used in the detection of metal ions, such as Cu^2+^, Hg^2+^, Mg^2+^, and Zn^2+^ [32,33]. Similarly, quinine, found in the rhizomes of *Polygonatum verticullatum,* can be used as a fluorescent tracer and is used to estimate a range of soil surface conditions and environments [32,34]. In addition, it was also used as an antimalarial agent and was the first fluorescent compound to be discovered; it was discovered by John Frederick William Herschel in 1845 after he noticed the emission from an aqueous quinine solution. Since then, it has been used as a fluorescence standard [35]. In the same way, the photophysical properties and applications of the sixty fluorescent compounds are shown in Table S1, the chemical structures of the fluorescent compounds reviewed in this article are shown in Figures S1 and S2, the structures of sixteen important naturally fluorescent compounds and their applications are listed in Table 1, and their chemical structures are shown in Figure 1. The sixteen compounds were selected based on their exceptional photophysical properties, specifically their quantum yield and their maximum applicability in different applications.

## 3. Chemical Structure-Driven Fluorescence Properties

Altering the chemical structure can optimize fluorescence properties. For example, the introduction of a long-chain alkyl group enhances optical properties and transforms the donor–acceptor interaction through intramolecular charge transfer (ICT), which regulates the substance’s sensitivity [10]. Similarly, varying the positions of substituents affects intermolecular interactions, imparting a stronger response to various triggers [56].

**Figure 2:**
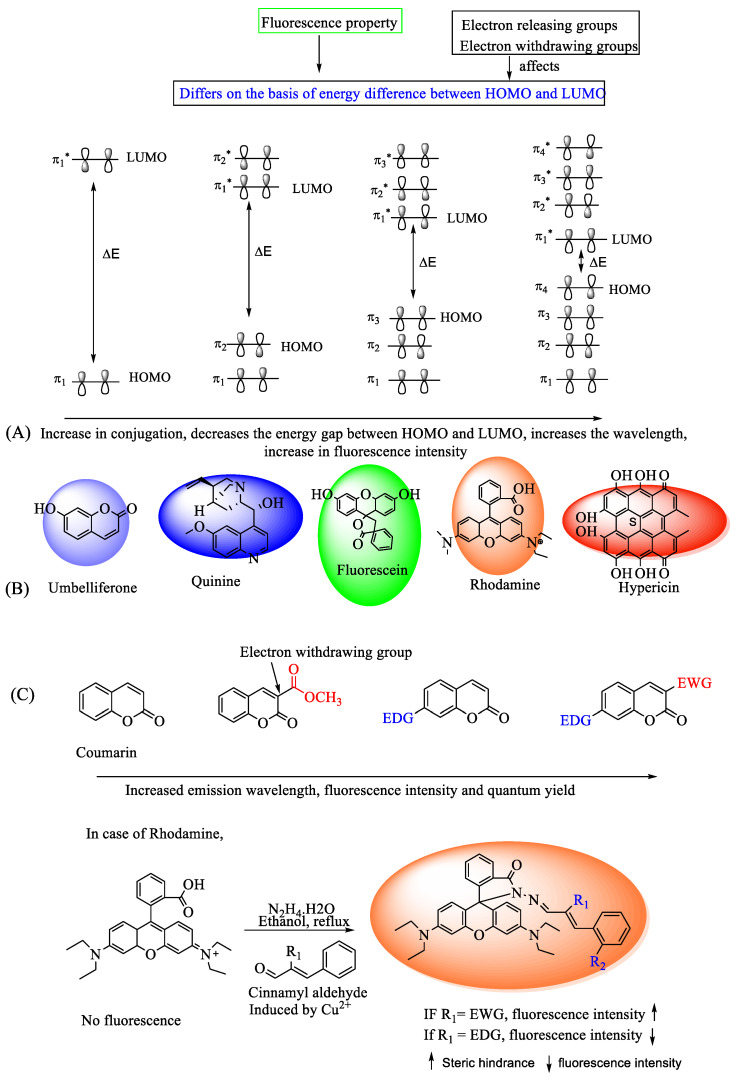
The structure–activity relationship of the fluorescent probes. (**A**) The effect of conjugation on the electronic excited state and its effect on fluorescence intensity—an increase in conjugation results in increasing fluorescence intensity. (**B**) Structures of different fluorescent compounds with their emission colors. (**C**) The effect of the electron-donating and electron-withdrawing groups on rhodamine’s intensity and quantum yield. * denotes the antibonding molecular orbital.

The biological activity of naturally occurring electrophilic substances, along with Michael acceptors, such as ring-strained forms such as lactones, lactams, epoxides, or other moieties, is the result of their inherent reactivity with cellular nucleophiles, such as cysteine, threonine, serine, or lysine residues, influencing the pathway of finding novel therapeutic targets [57]. Different small fluorescent probes impart different electrical and spectral characteristics and have been proven to have great selectivity and a variety of applications [58]. The spectral characteristics of fluorescent probes, including the wavelength, intensity, and photostability of the fluorescence emission, can be altered by changing the functional groups and molecular structure of the compound [59]. The absorption of UV or visible light by a conjugated π-system requires an energy-matching π-π* energy gap, enabling the excitation of a highest occupied molecular orbital (HOMO) π electron into the π* (antibonding) orbital or lowest unoccupied molecular orbital (LUMO) orbital. The conjugation of a compound does play a certain role in fluorescence emission. Increased conjugation leads to longer fluorescence emission, which often seems to be correlated with the linkage between alternate double and single bonds, which is related to the energy gap, as illustrated in Figure 2A [60]. Additionally, the presence of single-bond (sp^3^) carbon atoms influences the properties of conjugation through hyperconjugation, albeit to a lesser extent than conjugation and aromatic stabilization [61]. Figure 2B shows how different metabolites demonstrate different fluorescent emissions. However, there are conditions under which this relationship is exactly reversed, such as when a high proportion of the sp^2^ domain results in a shorter emission wavelength [61,62]. For instance, the extended amount of the sp^2^ domain in graphene-based materials might subject it to quantum confinement, resulting in the opening of a band gap [63]. This finding implies that other factors also play a role in controlling the fluorescence emission. The other factors might include the two common forms of the sp^2^ domain, the zigzag form and the armchair form. Even if the sp^2^ domains are the same, the armchair form’s edges are found to have shown short wavelength emissions, while the zigzag form’s ones show longer emissions [64].

The change in the photophysical properties due to differences in substitutions is very complex. It differs due to many effects, including the conjugation effect, steric effect, structure effect, and many more. There are instances in which the mobility of electrons is directly affected by different substituents; one which enhances the mobility will also increase the fluorescence emission, and vice versa [65].

In the study conducted by She et al., structure–property correlations were determined and the fluctuations in optical characteristics due to substituents imparting electronic effects and steric effects were examined in rhodamine derivatives [66]. The results revealed that, in comparison to electron-releasing groups, electron-withdrawing groups increased fluorescence to a greater extent, as shown in Figure 2C; however, this effect was not observed when the substituents were introduced at the meta- or para-positions of the benzene ring. Electron-withdrawing groups, like the nitro groups, affect the photophysical properties of fluorophores by increasing the delocalization of π-electrons in the aromatic ring [67]. When nitro groups are subjected para to the electron-releasing group, electron displacement occurs, resulting in a change in the difference between the HOMO and LUMO, which, in turn, changes the dipole moment and affects the photophysical properties, as in 4(5)-nitroimidazole and 5(6)-nitro benzimidazoles. Furthermore, the brightness of chromophores that consist of both electron-releasing and electron-withdrawing groups is enhanced by polarizing the π-system. Additionally, when a larger alkyl group is added to the side chain of the amino acid moiety of the fluorescent brightening agent, the fluorescence intensity of the compound increases [68]. Furthermore, the fluorophore’s brightness and absorption/emission wavelengths can be significantly impacted by the enclosing cavity of the fluorophore because of the creation of a solvation shell [69]. The creation of aggregates enhances the sensitivity of excited-state intramolecular proton-transfer (ESIPT) emissions. Attaching the extra fluorescent group to an ESIPT with fluorescence (or Förster) resonance energy transfer (FRET) allows for the development of longer-wavelength, near-infrared probes, which may detect physiologically relevant analytes in vivo [70]. Moreover, the photophysical properties and other fluorescence characteristics vary in terms of photobleaching, self-quenching, photostability, and solubility [71]. Certain carbocyanine dyes have the potential to self-quench, so changes to the carbocyanine structure lead to molecules that glow significantly on proteins, nucleic acids, or other biopolymers, comparable to cyanine dyes with incredible photostability [72]. Hence, the structure of a compound has a crucial relationship with its photophysical properties, and consequently, in biomedical applications, Figure 2 shows the relationships between the different substituents and structures and their activity.

## 4. Applications for Natural and Synthetic Fluorescent Compounds

Fluorescent probes and organic dyes derived from natural products and their synthetic analogs have immense potential in biomedicine, environmental monitoring, and food chemistry for detection and imaging, including ion monitoring, physiological and pathological processes in living organisms, tissue penetrations, and in vivo biomarker multimodal imaging [73]. In this section, the applications of fluorescent natural products and their synthetic analogs are mentioned and categorized into biomedical and analytical.

### 4.1. Biomedical Applications

**Figure 3:**
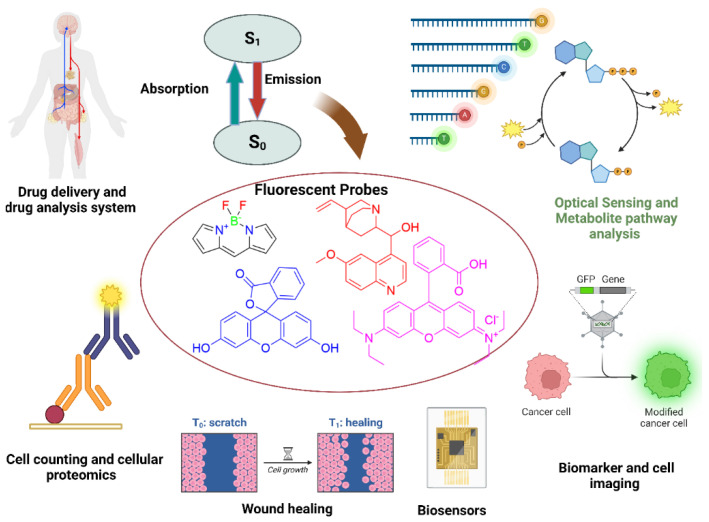
Schematic representation of biomedical applications of fluorescent probes. These applications are mainly based on the phenomenon of fluorescence, where the emission of radiation from the singlet excited state to the singlet ground state produces fluorescence and this relates to different utilities in drug delivery systems, cell counting and cellular proteomics, wound healing, biosensors, and biomarker and cell imaging. S_1_: singlet excited state; S_0_: singlet ground state; GFP: green fluorescent protein.

Fluorescent compounds have extensive biomedical applications, particularly in cell imaging, biosensing, drug discovery, in vivo imaging, medical diagnostics, therapeutics, and theranostics, as shown in Figure 3. Therefore, natural and synthetic fluorescent compounds span a wide field of research, from basic research to clinical diagnostics and therapy [73]. These compounds are extensively used in biomedicine due to their biocompatibility, specificity, sensitivity, low toxicity, good water solubility, and eco-friendliness; for example, the use of 1,8-naphthalimide, perylenediimide, morpholine, or N-methylpiperazine improves water solubility, making them appropriate for biomedical applications [74,75]. Moreover, in biomedicine, small fluorescent probes are frequently utilized in processes such as ICT, photoinduced electron transfer (PET) [76], ESIPT, stimulated emission depletion (STED) [77], FRET [78], fluorescence correlation spectroscopy (FCS), twisted intramolecular charge transfer (TICT), multiphoton fluorescence, and electron donor–acceptor phenomena [58].

The following are some biomedical applications of fluorescent natural products:

#### 4.1.1. Drug Delivery Systems and Drug Analysis

**Figure 4:**
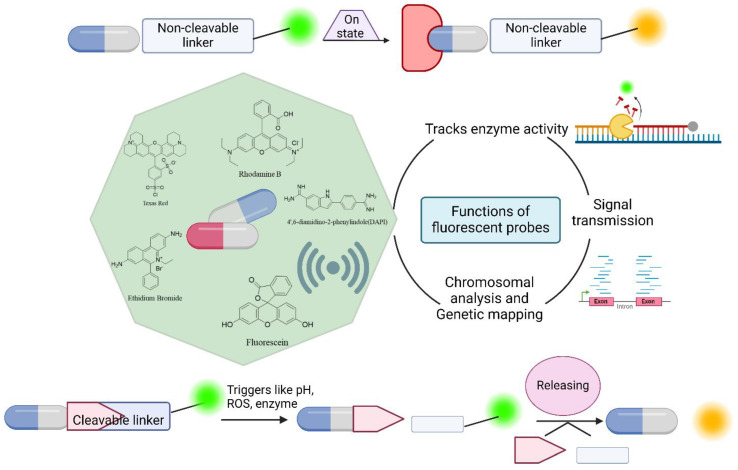
The working mechanism of fluorescent probes in drug delivery systems, drug analysis, and their function as sensors. Drugs attached with probes through cleavable linkage or non-cleavable linkage allow us to evaluate its delivery system properly, and its other function as a sensor helps us to analyze the metabolite pathway, enzyme activity, chromosomal analysis, and genetic mapping. The green color attached to the linker demonstrates the state before the drug is delivered to the target site, while the yellow color signifies the successful delivery of the drug to the desired place.

The use of fluorescent compounds as probes is crucial for monitoring real-time drug delivery systems. These compounds serve as valuable tools for tracking drug distribution and movement within the body, providing essential information that enhances the standard of medical treatment [79]. In the drug delivery process, a fluorescent probe is attached to the drug either through a biodegradable linkage or through a nondegradable linkage to form cleavable conjugates or non-cleavable conjugates, respectively, and the cleavable form exhibits dramatic changes in spectral properties when the bond is broken. In contrast, non-cleavable conjugates do not break down, even during the drug delivery process [80]. Under these conditions, the drug is not released because it is in the “off” state, and upon the discharge of the medication, the dye produces a fluorescence signal that transforms into an “on” state, as shown in Figure 4 [81]. Therefore, the real-time study of receptor kinetics during drug delivery is made possible by the application of GFPs. Using fluorescence-activated cell sorting (FACS), the released fluorescence may be identified, which can be used to sort cells based on the biomarkers of interest. This analysis technique provides valuable information about cell biology without costly mRNA analyses, risky radiolabeled binding experiments, or staining processes [82]. Fluorescent labeling can help to monitor the drug delivery process by providing information about material transport and distribution throughout the body [79].

Fluorescent probes have also been found to overcome the problems that arise from the drug delivery process. For example, in the ocular area, fluorescent probes are used to cover the nanocarrier surface, allowing nanosystems to be used as light probes and allowing the formulation of strategies that comprehend the route by which nanocarriers enter the ocular lens [80]. Recently, Wang et al. developed a probe that couples fluorescent dyes and the protein human serum albumin and exploited the ability of the protein to bind the drug by studying the interaction of tamoxifen (a drug used for breast cancer treatment) with the probe [83]. This allowed them to study the movement and delivery of the drug, which, consequently, allowed them to quantify the amount of the drug within the cells. Therefore, they concluded that these fluorescent probes present encouraging opportunities for therapeutic medication dosage recommendations.

Currently, anticancer nanomedicines are efficient for chemotherapy, and fluorescent dyes are added to the surface of nanoparticles (NPs) to enable fluorescence tracing in cells [84]. For example, green fluorescent fluorescein isothiocyanate-grafted silica NPs were used for in vivo drug administration [85]. Furthermore, 1,8-Naphthalimide-based fluorescent probes can conjugate chemically with 3-hydroxybenzyl and are successfully used for the detection of tyrosinase enzymes [74]. This polyphenolic enzyme can be taken as an alarming point of many diseases, such as Alzheimer’s disease, Parkinson’s disease, and skin cancer [80]. Moreover, photodynamic theory is one of the alternative low-level direct strategies that consists of a photosensitizer; a light-activated drug that stimulates reactive oxygen species and free radicals towards the treatment site with much lower side effects [86]. Despite being advantageous, its use still raises many questions because of its limitations, including the low bioavailability, poor permeability, and consolidation [87]. So, in today’s context, fluorescent carbon dots (FCDs), nanoliposomes, tocosomes, and liposomes are effective in drug delivery. They are also considered theragnostic because liposomes function as troubleshooting and FCDs behave as an external guiding agent [88]. Liposomes and nanoliposomes can transport hydrophilic and hydrophobic molecules separately or concurrently when a synergistic therapeutic effect occurs in addition to amphiphilic substances. These lipid vesicles are biodegradable and biocompatible carriers that can deliver regulated and sustained medication release. This feature of the lipid vesicles allows for excellent contact with biomembranes, enhancing effective tissue and cellular metabolism [89].

#### 4.1.2. Optical Sensing and Metabolite Pathway Analysis

Optical sensors are among the most versatile platforms for personalized health parameter monitoring and can completely change the way certain diseases are identified and managed because of the correlation of the variations in light released following excitation with higher energy photons to the presence or absence of a target analyte [90]. Some commercially available synthetic dyes, such as propidium iodide and propidium azide, are applicable to perform cell viability tests. However, these dyes can be equally toxic, expensive and demonstrate photobleaching effects. Therefore, to overcome these problems, carbon dots from the leaf extract of *Prosopis juliflora* can be used for biological applications in cellular imaging studies [91]. Moreover, fluorescent dyes can be used to monitor enzyme activity, signal transmission, protein transport, cellular integrity, membrane mobility, exocytosis, and endocytosis, along with chromosomal analysis and genetic mapping [6,92]. Natural compounds such as quinidine, quinine, cinchonine, and cinchonidine were presented as combinatorial fluorescent molecular logic gates in a study conducted by Agius and Magri [93], and these fluorescent logic gates can be used as diagnostic criteria for elevated chloride levels in body fluids [94]. Some natural and unnatural amino acids also exhibit various fluorescent properties based on the attachment with substituents at various positions on the amino acid [95,96]. These are widely used in modern cell biology, such as for gene encoding and the designing of novel artificial proteins, along with the evaluation of cell properties using optical imaging, dynamics, folding, and biomolecular interactions in proteins [97]. Each enzyme carries out its function, and any changes in its activity invite various serious diseases; therefore, the development of fluorescent probes that permit real-time analysis, allowing the straightforward and harmless observation of enzyme activity, might help in analyzing the causes of disease by elucidating the metabolic pathway [98]. It is easier to comprehend the functions of a cell when metabolic activity in the cell is detected. A fluorescent probe, coumarin with an N-(2-hydroxyethyl) carboxamide group at position 3, is capable of the fluorescence imaging of fatty acid beta-oxidation activity in the mitochondria of live cells [99]. Thus, fluorescent compounds can be used for the real-time analysis of the concentrations of metabolites and can be used for understanding metabolic processes.

#### 4.1.3. Biomarkers and Cell Imaging

A biomarker is an indicator of a disease or condition typically expressed in the form of biological change, biochemical activity, or cellular processes that can be detected through various physical, chemical, and biochemical methods. Biomarkers can be identified and measured from cell-conditioned media, bodily fluids, tissue specimens, and physical measurements of living systems [100]. They play an important role in the early detection of diseases. Here, fluorescent probes can identify targets and provide detectable signals indicating aberrant activity [98]. They are instrumental in monitoring the progression of diseases such as Parkinson’s disease, which is characterized by the unusual build-up of syn-nuclei, oxidative stress due to monoamine oxidase-B (MAO-B)-catalyzed oxidation, impaired mitochondrial function, and neurotransmitter deficits [101]. To detect this change, different fluorophores, such as coumarin, thioflavin-T, hydroxychrome probes, benzothiazole, benzylidene-imidazolinone, and benzofuranone-based probes, are used. These probes serve as biomarkers by using different mechanisms, including ESIPT, FRET, protein labeling, PET, ICT, structural rearrangement, and conformation modification strategies [70].

**Figure 5:**
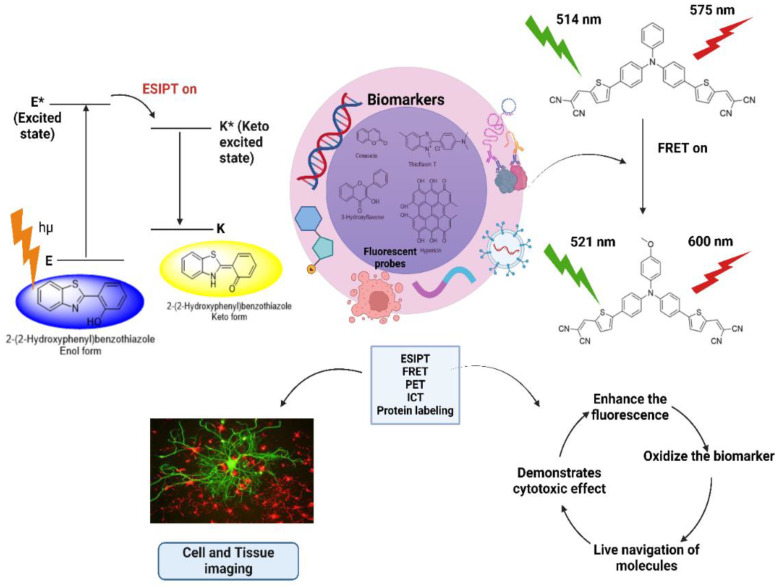
Mode of action of fluorescent probes in biomarkers and cell imaging. Fluorescent probes undergo different mechanisms, such as ESIPT, FRET, PET, ICT, and protein labeling, to intensify the fluorescence properties, followed by real-time analysis of molecules, thereby aiding in cell and tissue imaging. ESIPT: excited-state, intramolecular, proton-transfer (ESIPT) emissions; FRET: fluorescence (or Förster) resonance energy transfer; PET: photoinduced electron transfer; ICT: intramolecular charge transfer. * denotes the excited state.

ESIPT involves hydrogen bonding between proton donors, such as -OH, -SH, and -NH_2,_ and proton acceptors, such as -C=O and C=N [102]. When light is imparted, the ESIPT sources absorb radiation, jump to a more excited state, and create a photoisomerization phenomenon to create the excited state of its tautomeric form (Keto-enol tautomers). The electron-withdrawing nature of the keto form causes the decay of the excited state, the longer fluorescence is produced, and biomarker detection makes significant use of this phenomenon, as shown in Figure 5 [70]. The main strategy for using an ESIPT-based fluorophore is to modify the ESIPT process by first replacing the proton of the proton donor, due to which a short wavelength of the probe from the enol form is released. After interacting, a particular biomarker may initiate the ESIPT process to produce long-wavelength light, which produces different fluorescence [103], aiding in biomarker detection.

Odyniec et al. created a variety of 3-hydroxy flavone (3-HF) ESIPT boronate fluorescent probes to detect ONOO^−^ to observe various forms of amyloid-β aggregation [103]. Similarly, research carried out to determine the efficiency of a new fluorophore, Pl-Cys, a unique ESIPT dye connected to an acrylate group through a hydroxyl moiety, revealed that in the presence of Cys, the acrylate moiety splits off from Pl-Cys and Cys and, in turn, interacts with Pl-Cys, triggering the ESIPT phenomenon. This consequently imparted 20 times more fluorescence, which could aid in the detection of various diseases [104].

Biomolecules in living systems can be modified to exhibit fluorescence, which can facilitate early cancer detection by serving as biomarkers. Biomarkers linked with cancer can be identified based on the abnormal nature of DNA (DNA tetrahedron as a nanotweezer for the detection of cancer-linked mRNA), and aptamers or antibodies conjugated tetrahedron nanostructures (proteins, RNA, lipids), which specifies cancer states [105]. Dual-emission ratiometric fluorescence probes are extensively used in the bioimaging of biomarkers for accurate, quantifiable, and real-time studies [106]. This probe allows us to observe the variations in distinct fluorescence color types that are even visible to the naked eye and correspond to changes in content in detection systems, which helps in easy, quick, and effective target identification [107]. For example, a coumarin–benzopyrylium-based, ratiometric, fluorescent probe with a near-infrared turn-on has been utilized for the detection of Cu(II) at the subcellular level in living cells [108]. Live surgical molecular navigation, which uses fluorescently labeled markers, allows tumors and nerves to be displayed in real-time in contrasting pseudocolors [4]. Similarly, sialyl Lewis X (sLeX), a terminal O-glycan structure abundant in cancer cells, can be detected using a diboronic, fluorescent (DBF) probe, which acts as a noninvasive detector of prostate cancer cells [109]. Figure 5 demonstrates the mechanism of fluorescent probes in biomarkers and cell imaging.

##### Fluorescent Probes Aiding in Early Cancer Biomarker Detection

The early detection of cancer biomarkers can be crucial for the patient’s prognosis and aid in increasing the therapeutic efficacy of the treatment because a late-stage diagnosis adds complexity to the treatment [110]. The most commonly used methods for the detection/diagnosis of cancer include biopsy, radiography, genetic testing/profiling, diagnostic imaging, and endoscopic examination, which require a good sample quality and repeated testing. These techniques take a lot of time for the diagnosis and, thus, the chances of successful therapy will decrease. Hence, the efficient choice for detection would be the method that can detect the early stages of abnormality/changes in normal healthy cells as a result of the expression of biomarkers in the form of hormones, proteins, enzymes, and sugars [111,112,113,114]. The vision of researchers regarding creating a world where cancers are detected at the earliest stage before turning into complex and untreatable problems has driven them to explore new techniques that are not based on repeated testing and surgical intervention. They looked for the means to identify biomarkers that could indicate the presence of cancer, such as hormones, enzymes, proteins, and sugars, before a tumor gets too big. As they dug deeper, they discovered that the major biomarkers for cancers can be detected in circulating tumor cells (CTCs), nucleic acids, circulating tumor DNA, exosomes, and micro-RNA [115]. Among all of these, the fluorescence-based detection tools and technology can be decisive as they offer numerous advantages like rapid detection, noninvasive techniques, and the early detection of biomarkers with high sensitivity and selectivity due to the designing of selective probes for different types of biomarkers [110,116].

Epithelial cell adhesion molecule (EpCAM) has a strong expression on the surface of many epithelial cancer cells and, therefore, has been employed as a marker for CTCs [117]. Li et al. used a dual-component antibody system with EpCAM and folate receptor alpha (FRα) conjugated to Alexa Fluor^®^ 488 and DAPI to detect CTCs in peripheral blood samples. Furthermore, a surgical approach has been developed utilizing the near-infrared fluorescence dye IRDye800CW conjugated to an EpCAM antibody for the targeted visualization of tumor cells [118]. This conjugation highlights the potential of EpCAM towards the effective visualization of various tumors during surgery. Mucin 1 (MUC1) is another surface protein involved in tumor cell proliferation and Chen et al. developed CdTe quantum dots combined with aptamers to detect CTCs using MUC1 as a cancer biomarker [119]. Similarly, Kim et al. combined quantum dots with a siRNA delivery system to target cancer cells, creating a tool that serves as both a bioimaging agent and a gene delivery system [120].

Moreover, BODIPY-based probes also can distinguish different biomarkers. Wu et. al. designed and developed the deep tissue imaging BODIPY-based, near-infrared-I (NIR-I), noninvasive, small fluorescent probes for the in vivo detection of early breast cancer bone metastasis [121]. In their study, they developed the pH-activatable NIR probes as most of the cancer cells favor acidic environments and achieved the imaging up to 8 mm of tissue depth. Similarly, Tian et al. have developed the BODIPY-based, ratiometric fluorescence sensor for the selective detection of Homocysteine from cysteine and Glutathione which can offer intracellular quantitative analysis of Homocysteine in real-time imaging in neurons, as it is crucial for maintaining the redox homeostasis and its imbalance is associated to neuropathology disorder [122].

Rhodamine-based probes have the maximum potential to be used in detecting biomarkers because of their remarkable photophysical properties, such as large absorption coefficients, remarkable fluorescence, and quantum yields, especially NIR rhodamine probes [49]. A π-conjugation system of a rhodamine-based, near-infrared fluorescence probe was developed to detect the overexpression of the nitro reductase (NTR) enzyme in hypoxic tumor cells, which offered 32-fold fluorescence intensity enhancements and a low detection limit of 1.09 ng/mL, demonstrating the exceptional capability of rhodamine-based probes [123].

Li et. al. developed the FRET nanoprobe (HMSN/DOX/RVRR/PAMAM/TPE) by combining the properties of agglomeration-induced emission fluorophore and FRET pairs with the quantitative detection of the overexpression of Furin in the triple-negative breast cancer cell line (MDA-MB-468 cell) [124]. Here, they used hollow mesoporous silica nanoparticles (HMSNs) to load the antitumor drug doxorubicin (DOX), and the holes or nanopores were blocked or encapsulated by the Furin-specific responsive Arg-Val-Arg-Arg (RVRR) peptides and PAMAM/TPE. The FRET effect, in this case in cancer cells, is generated by the cleavage of the RVRR peptide by Furin, resulting in the release of doxorubicin.

#### 4.1.4. Biosensors

In 1956, Leland C. Clark, Jr., reported the first biosensor for the detection of oxygen, which led to a new development in the heart–lung devices used during cardiopulmonary bypass operations [125]. The International Union of Pure and Applied Chemistry characterizes a biosensor as a chemical sensor that combines a physicochemical sensor with a biologically derived identifying element [126]. The fluorescent protein sensor has applications in different areas, such as detecting pathogens and monitoring intracellular signaling molecules, and its development has been in process for more than 150 years [127]. There are many types of fluorescent biosensors, such as nucleic acid biosensors, DNA–RNA biosensors, quantum dot (QD)-based biosensors, fluorescent protein sensors, organic dyes, and NP-based biosensors [128]. Fluorescence-based biosensors can be made via natural or synthetic methods, which are particularly applied in drug discovery, food safety, disease diagnosis, environmental analysis, and bioimaging. In addition, fluorescent proteins, known as GFPs or synthetic fluorophores, have been extensively used in protein engineering [129]. They have also been used in probing the molecular dynamics of macromolecules, metabolites, molecular processes in living cells, and ions [130]. Traditional sensing techniques lack cellular and subcellular specificity; thus, new techniques are needed to directly quantify the dynamics of target molecules in specific cells and subcellular compartments [131]. The binding affinity, recognition element for a sensory domain, rate constant, choice of fluorophore, absorption and emission wavelength, and resolution of sensors are some of the factors that play important roles in developing biosensors [78]. Chelly et al. developed a fluorescent probe from the medicinal plant *Lavandula multifida* and used it as a fluorescent sensor due to its high stability and sensitivity for detecting metal ions, especially Hg^2+^, in marine water [132]. Similarly, Guisán-Ceinos et al. synthesized 3-azo conjugated BODIPY dyes to depict hypoxia-like situations in live cells, and this probe allowed the visualization with strong absorption and large molar absorption coefficients [133]. While synthetic fluorescent probes impart strong fluorescence, with enhanced other properties, fluorescent natural products have lower toxicity, environmental sustainability, and greater biocompatibility, thereby demonstrating their potential to be used as biosensors with diverse applications.

#### 4.1.5. Cell Counting System and Cellular Proteomics

The accurate quantification and characterization of cells are essential for medical diagnoses and disease treatment. There has been tremendous progress in medicine in terms of analyzing blood cells, performing cell therapy, evaluating the concentration of bacteria and viruses, and calculating the number of dead to live cells to measure the viability of cells with the aid of fluorescence [134]. Techniques such as fluorescence-based cell counting and viability assessments utilizing genetically encoded fluorescent sensors within cells have contributed to this tremendous progress [135,136]. More advanced methods of blood cell counting have produced more reportable parameters, which have been included in the regular complete blood count within the last few years [135], and these fluorescent sensors, genetically defined in a DNA sequence, are produced within the cells and are predominantly protein-based probes, using at least one fluorescent protein acting as a fluorophore [136].

One of the developing fields in cell biology is cell proteomics, which aims to create tiny molecule probes to comprehend the function of proteins by analyzing the target that binds to proteins, along with the molecular probes derived from nature [137]. Various fluorescent probes can covalently attach to surfactants, phospholipids, proteins, and polynucleotides, providing various types of information. They facilitate protein tagging by labeling reagents with appropriate functional groups, along with fluorescein, rhodamine, and erythrosin derivatives, because of the possibility of covalent bonding between amino groups and sulfydryl groups [6,138]. *L*-phenylalanine, *L*-tyrosine, and *L*-tryptophan are examples of naturally occurring amino acids with aromatic side chains that have been utilized as intrinsic fluorescence probes to investigate processes such as protein dynamics [6]. It is possible to directly and easily synthesize optically pure, fluorescent amino acids using inexpensive natural amino acids as the starting material. For instance, using the solid-phase approach, the cyano derivative of *L*-phenylalanine, a conformationally defined fluorescence and IR absorption probe, was utilized to research peptide membrane interactions, showing its sensitivity as a microenvironment indicator [139]. Similarly, the *L*-phenylalanine-based, genetically encodable, cyan-emitting fluorescent α-amino acids, such as 4-phenanthracen-9-yl-l-phenylalanine (Phen-AA) and 4-dibenzothiophen-4-yl-*L*-phenylalanine (DBT-FAA), have been synthesized and utilized for imaging at the cellular and sub-cellular level in live Hela cells [140,141,142]. Moreover, tyrosine-derived, fluorescent, unnatural amino acids were synthesized through methods such as the Heck coupling reaction, which have proven useful tools for the synthesis of novel fluorescent amino acids and can be incorporated into peptides for biological studies, including the examination of cellular functions, investigations of biological mechanisms, and the fabrication of optoelectronic devices [143]. Tryptophan (Trp), a fluorophore found in proteins, is naturally environmentally sensitive and has been shown in numerous instances in the literature to change its fluorescence spectrum upon substrate or ligand binding [144]. Similarly, the fluorescent amino acid *L*-(7-hydroxycoumarin-4-yl) ethylglycine, which is closely similar to tryptophan, has been incorporated into proteins as a probe for the urea-dependent denaturation of holomyoglobin [145]. Therefore, these types of natural, fluorophore-based fluorescent probes are significant for marking the potential functions of proteins and adding value to the cell counting system.

#### 4.1.6. Application for Wound Healing

Wound healing is a complex series of conserved evolutionary processes, including blood clotting, inflammation, cellular proliferation, and the remodeling of the extracellular matrix (ECM) [146]. Hemostasis, the first phase of wound healing, occurs within the first minutes to hours after injury and involves forming a platelet plug and releasing growth factors and immune mediators as distress signals to initiate repair. The inflammatory phase overlaps with the initial hemostasis phase during the first 72 h following injury. During this phase, molecular signals initiate neutrophil and monocyte infiltration to the injury site to eliminate further damage and clear foreign debris. Proliferation occurs between 4 and 21 days and involves re-establishing vascular and granulation tissue and re-epithelialization. New blood vessel formation occurs during this phase, and a number of chemical signals are emitted, including EGF, b-FGF, TGF-α, TGF-β, and VEGF [147]. The last phase is the remodeling phase, which occurs weeks after the initial injury and continues until scar tissue is formed. The differentiation of myofibroblasts drives remodeling in response to wound contraction. The four stages of wound healing are aided by the proteins and chemical signals produced by each type of cell [148].

The development of new biotechnologies will help us further understand, diagnose, and treat wounds. During wound healing, cell signaling molecules, including growth factors, chemokines, and cytokines, as well as products of cellular activity, are released into the ECM. Currently, the evaluation of wound healing mainly focuses on the quantitative detection of changes in wound size and granulation tissue formation. However, this method does not reveal what occurs at the molecular level. Luo et al. developed a NIR fluorescent probe, DCM-H_2_O_2,_ that can act as a diagnostic tool for chronic wound healing and for assessing fluctuations in H_2_O_2_ in chronic wounds in mice and humans [149]. The probe is synthesized from a penta fluorobenzene sulfonyl ester group as a recognition moiety and dicyanomethylene-benzopyran (DCM) as an NIR fluorophore. This response leads to a large Stokes shift, which emits a fluorescence signal at 695 nm. In another study, Nasir et al. tracked daily wound re-epithelization changes in human wounds over 6 days with the cell tracker dye 5-chloromethylfluorescein diacetate (CMFDA) after developing a serum-free ex vivo human partial thickness wound model. Human skin was obtained from human donors, and the subcutaneous fat was removed [150]. The fluorescent dye was added to the skin model for 30 min before it was washed with a PBS. Imaging was performed with an upright fluorescence microscope, wherein the area of the initial wound and the area of the open wound remaining were calculated. The calculations were used to determine the total epithelial wound closure. While their study was only performed utilizing 5-Chloromethylfluorescein diacetate (CMFDA) and cell tracker red CMPTX, there is a possibility of using other dyes or combining multiple fluorescent dyes to visualize cell migration patterns during wound healing. One of the limitations of using fluorescent dyes for the visualization of wounds during the study was the observation of irregular healing and how to interpret the factors contributing to that observation [150].

Irregular wound healing can be caused by the infiltration of bacteria. Microbial infection in wounds is a severe issue that can interfere with healing. The real-time monitoring and early detection of infections can be helpful in the treatment of wounds. Fluorescence imaging is a method used for bacterial visualization in wounds. The implementation of fluorescent imaging was associated with a 49% decrease in antibacterial dressing prescription and a 23% increase in wound healing rates, likely due to the early detection of bacterial infection, which leads to better hygienic treatment [151].

To determine both the type and location of pathogens, the MolecuLight i:X handheld device was developed for a quick diagnosis using fluorescent light illuminated by pathogenic bacteria. Moscicka et al. obtained fluorescence images using MolecuLight, where the bacterial load was comparable to that obtained from a microbiology laboratory [152]. The use of fluorescent biosensor devices allows for the early identification of bacterial invasion, which is critical for rapid treatment.

**Figure 6:**
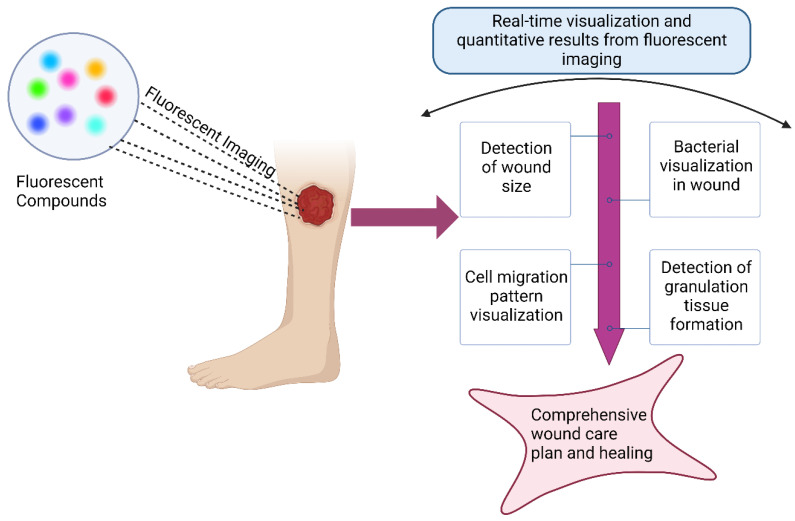
Application of fluorescent compounds in wound healing. Fluorescent compounds allow the real-time visualization and analysis of wounds, leading to an understanding of the healing process.

Noninvasive fluorescence imaging is used to visualize wound tissue at the point of care (POC). While MolecuLight i:X can improve the diagnosis of infected wounds, fluorescence imaging does not distinguish whether the bacteria are planktonic or in biofilm form. Le. et al. investigated the diagnostic accuracy of POC fluorescence imaging and compared the results to clinical results obtained during routine wound healing assessments [153]. They found that 287 out of 350 wounds had bacterial loads, and clinical assessment missed 85% of the lesions. Fluorescence imaging significantly increased the detection rate of bacteria. The use of fluorescence imaging provides an improved assessment of the wound state in real-time, compared to clinical assessment. These devices can be used for prompt detection using fluorescence, thereby expediting treatment to reduce the bacterial burden and facilitate healing. A schematic representation of the ability of fluorescent compounds to analyze and visualize bacteria in wounds is shown in Figure 6.

Smart bandages have emerged to provide the physiochemical surveillance of wound healing in real-time, as well as treatment. The bandages utilize smart sensors to detect wound infection. Zong et al. designed a series of smart bandages used to accelerate diabetic wound healing and provided the real-time fluorescent visualization of hypochlorous acid (HClO) levels. HClO levels are indicative of oxidative-stress-related inflammation and delayed wound healing [154]. In their study, SCy-7, an NIR fluorescent probe, was functionalized with a polymeric, ionic liquid that was cross-linked with hyaluronic acid. This probe was used to develop a fluorescence-based hydrogel wound dressing. Compared with the commercial Tegaderm™ film, the PIL-HA wound dressing was able to generate a fluorescent signal for the real-time quantification of HClO levels to monitor wound repair in real-time and treat the wound at the same time.

#### 4.1.7. Fluorescent Agents as Targeting Ligands in Tumors

Fluorescent agents can be incorporated with biological markers, like peptides, enzymes, antibodies, or bioactive compounds, which can display tumor signals that are specific to tumor cells [155]. Fluorescent agents, especially those with near-infrared fluorescence, like heptamethine cyanine, folic acid, fluorochrome, 6-substituted pyrrolo (2,3-d)pyrimidine antifolates, MHI-148, 5-aminolevulinic acid (5-ALA), and indocyanine green, are used for the diagnosis of tumors because of their unique and compatible properties, including high extinction coefficients, intense emissions, and huge Stokes shifts [156,157,158]. Amongst them, indocyanine green, 5-ALA, and methylene blue are used for the real-time analysis of tumors and are also considered non-targeted fluorophores, which work via the slow build-up of fluorophores in the tumorous regions, thereby helping in the visualization during the perfusion of tissues [156]. On the other hand, targeted fluorophores are activated only in specific circumstances, like when they get attached to targeted tissue. This target might be intracellular (enzymes) or extracellular (like matrix metalloproteinases, Cathespins) [159,160]. These probes might aid in pinpointing the location of the receptors, evaluate the interaction of the receptor and the ligand, and examine the mode of action of medications [161]. Furthermore, they should meet certain photophysical rules, such as wavelength, brightness, biostability, photostability, particular tissue accumulation, and pharmacokinetics, to be successfully used as targeting probes [162]. The ligand, the fluorophore, and the connector or the linker are the major components of a receptor-targeted probe [161]. The fluorophores should be highly specific, producing intense fluorescence signals after activation to allow significant target-to-background ratios. For this, mechanisms like PET, FRET, and H-dimer formation play a huge role [159]. Becker et al. found that after incorporating cyanine dye with octreotate, the analog of somatostatin, it showed higher fluorescence in tumor tissue, and they deduced that the dye–peptide conjugate formed by the specific interaction of the ligand and the receptor can be used for the diagnosis of tumors [163]. Recently, Zhang et al. discovered a specific near-infrared probe, 6-substituted pyrrolo [2,3-d] pyrimidines, which showed a distinctive affinity towards folate receptors, demonstrating its ability for the diagnosis of tumors [164]. In addition, Tazwa et al. provide an excellent review of various cell- and tissue-specific fluorescent probes that use adenovirus-based probes in a promoter-dependent manner [165]. Furthermore, during clinical surgery, an array of fluorophores, namely, methylene blue and indocyanine green, which are synthetic, have been used for fluorescence-guided tumor removal after receiving approval from the US Food and Drug Administration for clinical use [166].

#### 4.1.8. Other Biomedical Applications

Fluorescent compounds are important biochemical tools for a variety of biological research applications, ranging from visualizing cellular structures to monitoring molecular interactions. Some specific tools, such as fluorescein isothiocyanate (FITC), have been widely applied to protein labeling, antibodies, fluorescence microscopy, flow cytometry, and immunohistochemistry [167]. BODIPY dyes, rhodamine dyes, cyanine 5, and cyanine 3 dyes are versatile fluorophores that have been used for labeling lipids, proteins, nucleic acids, and biomolecules; microarray analyses; and multiplexed imaging [168]. In addition, many fluorophores incorporating NPs have attracted attention for their applicability as biosensors; for instance, a glucose biosensor based on fluorescence was created using the ameliorating effects of silver NPs on cadmium–selenium quantum dots, which resulted in a ninefold increase in fluorescence [169]. The characteristics of fluorescent probes rely on a range of fluorescence techniques, focusing on those that have shown promise in biological sensing and imaging. Furthermore, this shows how fluorescent probes with optimized optical properties are useful for biological analyses [170].

### 4.2. Analytical Applications

**Figure 7:**
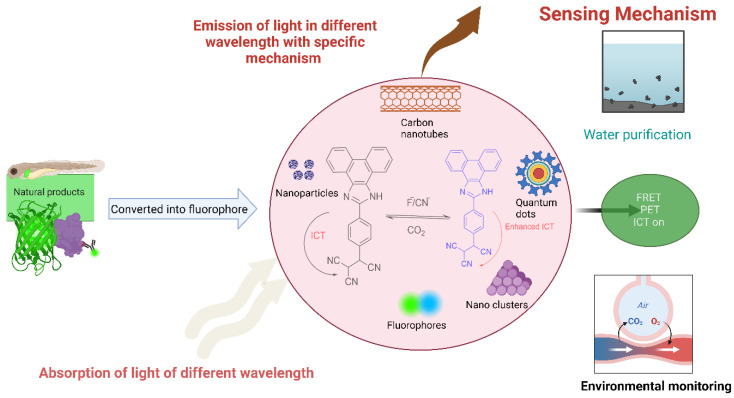
Schematic representation of the analytical applications of fluorescent probes. With the incorporation of fluorescent natural products into NPs, nanoclusters exhibit enhanced properties, increasing their utility in different fields, including water purification and environmental monitoring.

Due to their high sensitivity, selectivity, and ability to detect and quantify rare analytes, fluorescent compounds have wide-ranging applications in analytical chemistry, as shown in Figure 7 [171]. Many common analytical instruments, such as fluorometers, fluorescence microscopes, and plate readers, utilize fluorescence as their detection tool, such as when monitoring the concentration of various essential elements like Cu(II), Fe(II), Fe(III), and H_2_O_2_ (measure of oxidative stress) in live cells using confocal microscopy [108,172,173,174]. Fluorescence-based detection has been extensively used for the qualitative and quantitative analysis of environmental samples, pharmaceutical agents, nanomaterials, geochemical agents, atmospheric and interstellar molecules [169,175,176]. Some of the applications related to the analytical field are described below:

#### 4.2.1. Environmental Analysis

Environmental pollutants have become a major concern over the past decade, and these pollutants have numerous harmful effects, including carcinogenesis, growth retardation, endocrine disruption, etc. [177]. Fluorescent natural products can act as molecular probes to monitor changes, such as pH, temperature, and specific pollutants, and can aid in analyzing environmental problems [175]. They are instrumental in detecting heavy metal contamination and other chemical and biochemical pollutants [178]. For instance, an ethyl cellulose-modified fluorescent probe was used to detect Al^3+^ in the environment and food products, demonstrating a high sensitivity and effectiveness in mitigating the hazardous effects of aluminum [128]. In addition, fluorescent detectors in conjunction with chromatographic techniques have also been pivotal in analyzing contaminants in diverse environmental samples. Much research regarding the determination of polyaromatic hydrocarbons in water, soil, and sediments; pesticides in soil; and water using fluorescent detectors has been performed, and these detectors are effective at quantifying harmful contaminants [176].

With the use of a smartphone, Shang et al. used an infrared fluorescent probe for the detection of SO_2_ derivatives, HSO_3_^−^, in industrial effluent and food samples immediately and on the spot, which might help in comprehending the hazardous effects of SO_2_ derivatives on nature and living beings [179]. Similarly, Ali et al. developed an effective donor–acceptor ICT-type probe (tricyanoethylphenyl phenanthroimidazole) for the detection of CO_2,_ as shown in Figure 7, and this development of the probe might lead to understanding global climate change and greenhouse effects [180]. Moreover, a novel ratiometric fluorescent probe, PBQ-AB, with a large Stokes shift and emission wavelength shift, was developed for the real-time tracking of hydrazine in environmental soil and water samples, revealing the diverse applications of fluorescent probes in environmental analyses [181]. Therefore, fluorescent probes have been used as important substrates that play a crucial role in ecological analyses, offering sensitive and effective tools for examining and removing environmental pollutants.

#### 4.2.2. Water Purification

Improving the sustainable decontamination and purification of water is a major concern, and fluorescence spectroscopy has emerged as one of the most promising tools for characterizing organic matter in seawater and freshwater, monitoring diesel pollution, evaluating water treatment processes, detecting insecticides, and assessing the quality of raw sewage. Additionally, fluorescence spectroscopy can be used for discharge detection in wastewater treatment plants [182]. Nonbiodegradable waste such as antibiotics in water can be converted into nontoxic products such as water and carbon dioxide by photocatalytic reduction [183]. Similarly, a fluorescence excitation–emission matrix can be used to obtain valuable information on dissolved organic matter in water [184] and to evaluate the impact of wastewater on natural water, as the composition of effluent organic matter is different from that of naturally occurring organic matter (OM) [185].

Based on the classification provided by Li et al., the fluorophores in wastewater are divided into two groups: region Em > 380 nm (fluorophores with limited aromatic rings), and region Em < 380 nm (polycyclic aromatic rings) [186]. Similarly, the region λ_excitation_/λ_emission_ ~ 225/350 nm is considered as peak T, and λ_excitation_/λ_emission_ ~ 300–350/400–500 nm is considered as peak C. Phenols, DNA, aromatic amino acids, and lignin are the organic wastewater components falling in the region Em < 380 nm, and fluorophore-like humic acids, flavonoids, and quinones fall under the region Em > 380 nm [187]. The different contaminants present in domestic, industrial, animal, and pharmaceutical waste in water can be detected in these regions and can be used as a tool for the identification of the category of waste, which is crucial for waste management.

Likewise, the correlation between biochemical oxygen demand (BOD) and peak T provides a direct link to the microbial activity in this region, and peak T fluorescence is considered an indicator of the presence/absence of fecal contaminants and sludge systems [188]. Additionally, with this technique, both the online and offline monitoring of wastewater can be performed [189]. It can convert peak T fluorescence into BOD-equivalent values with the use of an internal calibration factor [190]. On performing real-time fluorescence with some conventional methods, the chemical oxygen demand (COD) can be determined for influent and effluent [191], and the efficiency of treatment methods can also be determined. The ratio of peak T to peak C fluorescence intensity indicated the presence of remnants of farm waste products in the water; hence, fluorescence spectroscopy could be a potential tool for plant- and animal-derived waste control [192]. Therefore, real-time fluorescence spectroscopy has potential for wastewater management in modern research. Further studies should be performed to determine the negative effects caused by pH, temperature, coagulation, and so on [193]. In summary, fluorophores have significant applications in modern wastewater management research; however, challenges still need to be addressed.

Fluorescence-based methods can be used to characterize structural materials, paints, artwork, and geological structures. Specifically, the chemical composition of the artwork of an organic material can be analyzed using fluorescence. X-ray fluorescence (XRF) is routinely used as an analytical method in geochemistry, wherein soil and sediments can be characterized with high accuracy [194].

## 5. Current Research Trends

### 5.1. Fluorescent Nanoparticles, Nanoclusters, Quantum Dots, and Carbon Nanotubes

The incorporation of natural-product-based fluorescent probes, such as NPs, nanoclusters, QDs, and carbon nanotubes, is a promising field of research. These small probes with special features, including their size, tunable optical properties, and biocompatibility, have emerged as remarkable approaches for various applications, including environmental sensing, chemosensing, drug administration, etc. [85,169,195]. Numerous fluorophores can be tagged and developed into NPs, concentrating the fluorescence signal in a specific region, thereby enhancing the brightness [196]. They are primarily used in biolabeling as alternatives to traditional organic dyes and have been developed as chemosensors and biosensors whose sensing mechanism relies on the ability of the substrate to either amplify or quench the fluorescence [197,198].

Carbon-based QDs are a novel class of optical nanomaterials that are attractive as fluorescent bioimaging agents due to their low toxicity, aqueous solubility, biocompatibility, high optical performance, and chemical and photochemical stability [199]. Fe_3_O_4_ nanoparticles coated by a matrix and amine-functionalized quantum dots as key parts of a multifunctional nanocarrier system in the nanosphere can be utilized for cancer diagnoses and targeted treatment by dual imaging systems: fluorescence and magnetic resonance [200]. Water-soluble carbon dots derived from bamboo leaves [201], saffron, and spices [202] are capable of emitting fluorescence when exposed to light. A growing number of FRET-based applications are using gold NPs (for sensing DNA) and semiconductor QD quenchers because of their remarkable quenching capacity, reduced background signal, enhanced sensitivity, and capacity to mark gold NPs and QDs with various physiologically active groups [170,195]. Similarly, photo-switchable fluorescent NPs are extensively used in biological labeling and the super-resolution imaging of cellular samples [170]. Because of their exceptional stability, distinct fluorescent properties, low toxicity, biocompatibility, and potential applications, Au nanoclusters are used for determining analytes in real samples, such as serums, lake water, and cultured cells; identifying and analyzing materials containing cations, anions, tiny chemical compounds, biomolecules, or hydroxyl radicals; and measuring intracellular temperatures [203]. Single-walled carbon nanotube (SWCNT) NIR fluorescence has been used to create optical sensors and monitor nucleic acids, proteins, and drugs by tracking the position and form of SWCNTs incorporated into living cells [204].

#### Role in Early Detection of Cancer

Functionalized fluorescent nanoparticles provide the greatest tool for the detection of cancer biomarkers. For example, Federica et al. developed a tool through which cancer-related micro-RNA (miR-203) can be quantified in real-time using a fluorescently labeled DNA probe on PEGylated gold nanoparticles (AuNPs) as a reporting technique for cancer biomarkers in clinical practices [205]. Similarly, oligonucleotide-modified gold nanoparticle probes hybridized to fluorophores and labeled as “nano flare” have been developed for the detection of mRNA in living cells [206]. Here, the fluorescence of the fluorophore is quenched by the Au NPs. However, in the presence of target mRNA, the oligonucleotide forms a complex, which then causes the release of fluorescent reporter sequences from the AuNP’s surface, resulting in fluorescent signals like flares. Moreover, these “nano flares” have been developed further by Tang et al. for the detection of the expression of multiple mRNAs simultaneously in a single-target assay [207]. Here, an AuNP core was hybridized with three short, dye-terminated reporter sequences, which detect the three different types of tumor-related mRNA targets; c-myc mRNA, TK1 mRNA, and GalNAc-T mRNA were determined by three different fluorescent signals, green, yellow, and red, respectively. In addition, the Au NP-based hairpin structures labeled with fluorescein, Texas red, and Cy5 were used as molecular beacons for the detection of three different tumor-suppressor genes [208]. Kim et al. combined quantum dots with an siRNA delivery system to target cancer cells, creating a tool that serves as both a bioimaging agent and a gene delivery system [120]. Dong et al.’s 2022 review article provides comprehensive information on various nucleic-acid-based nanosensors for tumor RNA monitoring in cancer diagnostics [209].

Extracellular vesicles are membrane-bound nanoparticles released by cells throughout the body, with typical sizes ranging from 30 to 1000 nm in diameter. Exosomes, a subclass of extracellular vesicles, are 30–150 nm in diameter. Exosomes and extracellular vesicles hold significant promises as cancer biomarkers and drug delivery vehicles. Recently, Chen et al. developed a fluorescence-based approach using PKH26, or PbS quantum dots, to stain small extracellular vesicles; this allowed the researchers to study vesicle circulation and biodistribution in major organs in a time-dependent manner [210]. Wu et al. recently developed a ratiometric fluorescence assay to detect plasma exosomes as a cancer diagnostic tool [211]. This assay employs a Cy5-tagged aptamer, targeted against the exosomal surface protein CD63. Previously, Huang et al. developed a fluorescence-based exosome biosensor aimed at gastric-cancer-derived exosomes, utilizing rolling circle amplification to enhance the signal, showing great promise for cancer diagnostics [212].

Hence, the process of incorporating fluorescent probes as NPs has created a wide range of applications.

### 5.2. Fluorescent Natural Products

Fluorescent natural products are being utilized in several ways in biomedical and bioanalytical research. Recently, the first sustainable, naturally derived, affordable luminous carbon dots based on two networked hydrogels (agarose and polyacrylamide) were created, which showed strong mechanical durability, resistance to stretching, self-healing capacity, and Fe^3+^ responsiveness. These can be used for creating luminous patterns and data encryption [213]. Researchers are interested in developing new fluorescent probes, followed by the characterization of their applicability in various fields, including biosensing, cellular imaging, and drug discovery [214,215]. In a recent study, Dazat et al. used fluorescent, natural, eutectic solvents (a mixture of fructose and urea, a mixture of citric acid and glucose, and many more) for the improvement of ergosterol detection in mushrooms to analyze fungal contamination in edible mushrooms [216]. Phytochromes are naturally occurring photoreceptors that are used as sensors because of their ability to respond to near-infrared light and control bacterial metabolism. Similarly, biliverdin, a naturally occurring byproduct of heme catabolism in human cells, serves as the chromophore in all proteins and can be used as a genetically encoded fluorescent probe [217]. Moreover, Liu et al. cataloged the inherent fluorescence of substances, such as oils, wines, and honey, as an innovative method of combating food fraud. They created a tiny library of fluorescence fingerprints by mapping the distinctive fluorescence of each product over a range of wavelengths, which helped in analyzing the quality of the product [218].

Many natural organophosphorus compounds (pentavalent and trivalent phosphanes, unsaturated phosphanes, and heterophospholes) are being used as optoelectronic materials in the field of electronics, such as field effect transistors, sensors, light emitting diodes, and polymers for organic photovoltaics [219]. In addition, the electrospinning method is used to prepare many fluorescent nanofibers made from thermosetting polymers, and it has shown significant fluorescence emissions, especially when the phenyl ring of a thermoplastic elastomer is confined by its intra- and intermolecular mobility [220]. Similarly, velocity-, pH-, and polarity (VPP)-targeted probes enable the real-time examination of pathogenic microenvironments, which yields vital insights for prompt diagnoses, process tracking, and the prediction of pathophysiological processes [221], but as of now, the use of natural fluorophores in tumor removal is limited and still in the research phase, hopefully to be successfully used for clinical applications. Natural-based fluorophores often get overshadowed by synthetic ones, or their derivatives, due to their greater fluorescence intensity and stability. However, the research into fluorescent natural products has seen significant advancements in recent years, and many researchers are still searching for fluorescent natural products that can aid in many different applications. Overall, the research into fluorescent natural products is vibrant and interdisciplinary, with ongoing efforts to explore their diverse applications and unravel their biological and chemical properties.

## 6. Opportunity and Challenges in Research

Current research into fluorescent probes has provided exciting opportunities for biomedical applications due to their unique properties, biocompatibility, high quantum yield, high specificity, sensitivity, and unique photophysical and photochemical properties. These qualities not only make them novel probes for biological imaging but also open possibilities for other applications like the structural and dynamic characteristics of macromolecules. In addition, they show wide potential for analyzing metabolic pathways, which can accelerate the discovery of new therapeutic agents for curing various diseases. The exploration of complex biological systems at the molecular level can also be performed using fluorescent natural products. In addition, their ability to be used in monitoring pollutants, toxins, and other environmental factors has demonstrated the vast potential of these products; therefore, these compounds can further be utilized for developing new materials with unique properties that can aid in different applications.

There are several challenges in the development of novel probes for fluorescence-based natural products, such as their isolation, characterization, synthetic accessibility, biological compatibility, sensitivity, and quantum yield, as well as their chemical and biochemical properties, such as pH, solvent polarity, temperature, cytotoxicity, pharmacokinetics, and pharmacodynamics. Furthermore, the isolation of the fluorescent product in its pure form remains a challenge because the concentration of the fluorescent natural products from complex biological samples is relatively low. In addition, the problem of accurately elucidating the structure of fluorescent natural products represents the next hurdle in the development of fluorophores. Similarly, the limited chemical accessibility and lack of highly active compounds also influence the development of fluorescent natural products.

Nonetheless, there is still much scope for natural fluorescent metabolites in many fields; one of the underappreciated and potential fields could be material science, in which some of the areas of application could be field effect transistors, light-emitting diodes, and many more, and these areas could further highlight the importance of natural fluorescent products. In the case of synthetic fluorescent probes, although they have a wide range of applications due to their stability, tailoring ability, and compatibility, the main challenges occur in terms of their toxicity, complexity, cost, and environmental sensitivity. Despite the challenges, there is significant potential for both natural and synthetic fluorescent probes across various fields, and advances in overcoming these challenges will foster their effectiveness.

## 7. Conclusions

This review highlights the wide-ranging applications of natural products and synthetic fluorescent compounds in biomedical and analytical research. We discussed the usefulness of fluorescent probes in cell imaging; biomarker discovery (primarily in cancers and tumors); optical sensing; wound healing; drug delivery systems; and analytical applications, including environmental analyses and water purification, and as targeting ligands in tumors. Furthermore, the structural and chemical properties of many natural products were discussed, with an emphasis on their photophysical and photochemical properties, including specific fluorescent properties. This extensive review demonstrates that fluorophores are emerging techniques for understanding biochemical systems that can be used for studying drug effects at cellular and subcellular levels. Fluorescent probes also help to elucidate pharmacokinetics and pharmacodynamics during the drug discovery process. Novel areas of application, such as wound healing, cancer diagnostics, and cellular proteomics, were further discussed. Recently, the rapid growth of fluorescence-based visualization and quantification techniques in both biomedical (imparting its prime role in cancers and environmental analysis has further inspired the development of new natural-product-based fluorescent probes) and synthetic analogs. Overall, this review will facilitate further research on the photophysical and photochemical properties of different fluorescent compounds that are applicable in biomedical and analytical research.

## Figures and Tables

**Table 1:** The list of common natural and synthetic fluorescents with general photophysical properties.

S.N.	Fluorescent Compound	Derivatives	Excitation Phenomenon	Photophysical Properties				Solvent	Biomedical Applications	References
				Absorption Maximum (nm)	Emission Max (nm)	Molar Excitation Coefficient (M^−1^ cm^−1^)	Quantum Yield (%)			
1.	Curcumin	mono-biotinylated curcumin	proton donor–acceptor phenomena	408–430	450–560	55,000	0.0104–0.174	Cyclohexane	It has a high fluorescence intensity; it helps researchers to understand the nature and membrane polarity of cells; it binds to the lipids of membrane and proteins of cells; it is useful for identifying the changes in the structure of the host.	[36,37]
2.	Xanthone	xanthone-2-carboxylic, dimethylxanthenone-4-arcetic acid	donor–acceptor phenomenon	338	355–400	6828	0.97	Acetonitrile	It is a photobase generation mechanism; it is used to monitor photocatalysis reactions; it has high photostability.	[38]
3.	Phycobili protein	B-phycoerythrin, allophycocyanin, phycocyanin, phycoerythrin	electronic excitation	495–655	580–660	1.9–2.41 × 10^6^	0.98	Aqueous	Bioimaging; the diagnosis of human metabolic disorder; chelating agents.	[39,40]
4	Perylene	Perylene-3,4,9,10-tetracarboxylic diimide (PTCDI),	donor–π–acceptor; strong π-π intermolecular interactions	440	444	34,000	0.95	Toluene	The study of the morphology of cells by entering without disruption.	[41]
5.	Umbelliferone (7-hydroxy coumarin)	4-methyl umbelliferone	proton donor–acceptor phenomena	323–370	396–480	12,500–21,500	0.30–0.87	Dimethylformamide, chloroform, water, ethanol, water with acid or base	It aggregates around bacterial and fungal regions and generates excited-state hydrogen transfer reactions, useful in photodynamic theory	[42]
6.	Quinine	quinolines	electronic excitation	350	450	1.09 × 10^4^	0.55	0.5 N Sulphuric acid	It is antimalarial and is a precursor for modern synthetic anti-plasmodial quinolines.	[43]
7.	Styrene	triphenylamine or carbazole-substituted styrene derivatives	conjugations phenomenon	426–518	538–606	29,400–40,300	0.02–0.037	DMSO	It has a high thermal stability, photoelectronic properties, and charges transport material.	[44,45]
8.	Fluorescein (derived from Xanthone)	carboxyfluorescein, fluorescein di-acetate (FDA), carboxyfluorescein di-acetate (CFDA)	electronic excitations	490	515	88,000	0.93	0.01 M NaOH	Cellular pH imaging; cancer therapy; markers for bacterial growth; and it has a dual sensor.	[38,46]
9.	BODIPY (inspired from porphyrins; derived from acetic anhydride and 2,4-dimethylpyrrole)	4,4-Difluoro-4-bora-3a, 4a-diaza-s-indacene	electronic excitations	505	515	>50,000	0.94	Toluene	It has tunable electronic and photonic properties and dye-sensitized solar cells (DSSCs).	[47]
10.	1,8-naphthalimide (derived from naphthalene)	4-N-methylpiperazine-1,8-napthalimide	conjugations phenomenon	398–416	494–525	(3.87–4.12) × 10^4^	0.011–0.594	Methanol	Antimicrobial, antibacterial, and anticancer agents.	[48]
11.	Rhodamine (derived from Xanthene)	Si-rhodamine,4-carboxyrhodamines	donor–acceptor of electrons	512	530	8.57	0.86	PBS	The labeling of intracellular structures; isomeric tuning; Halo-tagged and SNAP-tagged proteins.	[38,49]
12.	Carbostyril 124 (related to indoles and quinoline)	carbostyril (cs124,7-amino-4-methyl-2(1H)-quinolinone),	excitation and emission	348	381	16,000	0.97	Water	A small-molecule, organic antenna for Lanthanide-based FRET.	[50]
13.	p-Aminophenol (derived from phenol and aniline)	Fluorofluorophores (Chromene, Squaraine, Oxazine)	electronic excitation	363–641	435–664	1.9 × 10^4^–1.68 × 10^5^	0.03–0.89	Ethanol	They are used as fluorescent nanoemulsions; they hold promise as brilliant bioimaging agents.	[51]
14.	Squarylium dye III (derived from Squaric acid)	indodicarbocyanine dye D3, squarylium dye D1	donor–acceptor–donor of electrons	627.6	676	30,900	0.65	Methylene chloride	They are photosensitizer, biolabeling, and chemosensory materials for analytical uses in the biomedical field.	[52]
15.	Pyrene	1,3,6,8-tetrakis(4-n-monoethyleneglycol-(2-propynyl) ether) pyrene, 1,3,6,8-tetrakis(4-n-diethyleneglycol-(2-propynyl)ether)pyrene and	dipolar donor–π–acceptor (D–π–A)	378	422	100,000	0.99	Methanol	The detection of reactive oxygen species (ROS) due to strong emission of fluorescence in live cells.	[53]
16.	1,2,4-trioxolane	4H-1,2,4-triazole,4-alkyl-3,5-bis(4-bromophenyl)-4H-1,2,4-triazoles	π-conjugated systems	472	545	5 × 10^5^	>0.98	Dichloromethane	They are used for the detection of cell morphological changes; they are anticancer agents.	[54,55]

S.N. denotes the structure number of compounds.

## Supplementary Materials

The following supporting information can be downloaded at https://www.mdpi.com/article/10.3390/bioengineering11121292/s1. Figure S1: structures of some of the natural-based fluorescent compounds. Table S1: the list of common fluorescent compounds with general photophysical properties.
