# Supplementary material for: Advances in Natural-Product-Based Fluorescent Agents and Synthetic Analogues for Analytical and Biomedical Applications
Source: Bioengineering (Basel). 2024 Dec 19;11(12):1292. doi: 10.3390/bioengineering11121292 (PMC11727039; doi:10.3390/bioengineering11121292)
Supplement: Supplementary file 1 [file bioengineering-11-01292-s001.zip › bioengineering-3352326-supplementary.pdf]

Advances in Natural-Product-Based Fluorescent Agents and Synthetic Analogues for Analytical and Biomedical Applications

Soniya Joshi [1], Alexis Moody [2], Padamlal Budthapa [1], Anita Gurung [1], Rachana Gautam [1], Prabha Sanjel [1],
Aakash Gupta [3], Surya P. Aryal [4], Niranjan Parajuli [1,*] and Narayan Bhattarai [2,*]

[1] Central Department of Chemistry, Tribhuvan University, Kathmandu 44618, Nepal; soniyajoshi157@gmail.com (S.J.); padambudthapa8@gmail.com (P.B.); anitagurung9855@gmail.com (A.G.); rachanag759@gmail.com (R.G.); prabhasanjel01@gmail.com (P.S.)
[2] Department of Chemical, Biological, and Bioengineering, North Carolina A&T State University, Greensboro, NC 27411, USA; amoody@aggies.ncat.edu
[3] Department of Biomedical Engineering, University of Wisconsin-Milwaukee, Milwaukee, WI 53211, USA; gupta_aakash_007@yahoo.com
[4] Department of Chemistry, University of Kentucky, Lexington, KY 40506, USA; aryalpsurya@gmail.com
* Correspondence: niranjan.parajuli@cdc.tu.edu.np (N.P.); nbhattar@ncat.edu (N.B.)

Table S1: The list of common fluorescent compounds with general photophysical properties

S.N.	Fluorescent compound	Derivatives	Excitation phenomena	Solvent	Photo-physical properties				Applications	References
					Absorptions maximum(nm)	Emission max(nm)	Molar excitation coefficient (M^{-1} cm^{-1})	quantum yield (%)		
1.	Curcumin	mono-biotinylated curcumin	Proton donor-acceptor phenomena	Cyclohexane	408	446	55000	0.014	higher fluorescence intensity in cancerous cell than normal cells, helps to understand the	[1]

									nature and membrane polarity of cells, binds to lipids of membrane and proteins of cells, useful for knowing the changes in the structure in host	
**2.**	Xanthone	xanthone-2-carboxylic, dimethylxanthenone-4-acetic acid	donor-acceptor phenomenon	Acetonitrile	338	400	6828	0.97	photo base generation mechanism, moniter the photo-catalyisis reactions, photo stability	[2,3]
**3.**	Perylene	Perylene-3,4,9,10-tetracarboxylic diimide (PTCDI)**,**	donor-π-acceptor,trong π-π intermolecular interactions		438	530	38500	0.94	successfully enter in living cells without destroying their morphology, intracellular biomedical application	[4]

**4.**	umbelliferone	7-hydroxycoumarins, 4-methylumbelliferone 4-methyl-7 hydroxycoumarin	Electronic transitions	(H_2O + 95% H_2SO_4)	330	430	13300	0.81	Anticancer agent ,, 160 CYP genes coding for P450 cytochromes,blue-emitting dyes uoresce	[5]
**5.**	Phenoxazine	6,7-diphenyl-[1,2,5] thiadiazolo[3,4-g] quinoxaline -ph phenoxazine (DPTQ-phPXZ)	Electron donor-acceptor phenomena	Cyclohexane	850	768	23.5×10^3	0.155 (Measured in non-polar cyclohexane)	Anticancer drugs containing phenoxazine moiety binds to DNA at the transcription initiation complex and inhibits RNA polymerase from elongating the RNA chain	[6,7]
**6.**	Quinine	quinolines	electronic excitation	0.5 N H_2SO_4	350	450	1.09×10^4	0.55	anti-malarial and precursor for modern synthetic anti-plasmodial quinolines	[8]

**7.**	Coumarin	7-diethylamino-4-methylcoumarin, 4-Hydroxycoumarin	Electronic excitation	Methyl cyclohexane	373	445	2.35×10^4	0.50	protein labeling, molecular probes for bioimaging; water-soluble photostable, pH insensitive	[1]
**8.**	Griseofulvin	Griseofulvin-4'-alcohol (II)	Electron excitation	DMSO	295	420		0.108	Antiproliferative action on cancer cells of mammals	[9,10]
**9.**	Stilbene	1,4-bis-dodecyloxy-2,5-bis(2-{4-[2-(4-methanesulfonyl-phenyl-(vinyl]-benzene (tetra-stilbene)	Electronic excitation	Dichloromethane	421	517	8.7×10^4	0.13	Optoelectronic device with significant fluorescence emissions and high heat stability	[11]
**10.**	Phycobiliprotein	B-phycoerythrin, allophycocyanin, phycocyanin, phycoerythrin	Electronic excitation	H_2O	500–565	575	2.41×10^6	0.98	bio-imaging, diagnosis of human metabolamic disorder ,chelating agents	[12–14]
**11.**	Anthraquinone	Crinemodin Rhodoptilometrin		Methanol	452	534	9.0×10^3	0.6	accumulates outside of the	[15]

									nucleus in the cytoplasmic organelles, offers special starting points for structural alteration resulting in better imaging agents	
12.	Carbazoles	3-(6-benzothiazol-2-yl-9H-hexylcarbazole-3-yl)-2-cyano-acylic acid	Excitation-emmission	DCM	319	391	30125	0.057	Outstanding biocompatibility, high planarity, favourable donor ability, provides feedback to the small concentration changes of biomarkers at the site of infection	[16, 17]
13.	Quinoline	(2-cyano-3-(2,6-dimethoxyquinolin-3-yl) acrylic acid (Q1), 2-		Ethanol	393	472	2.27×10^{2}	0.83	photovoltaic cells, polymer solar cells,	[18]

		methyl-N,N-diphenylquinazolin-6-amine (Q5)							emission layer of organic light-emitting diodes (OLEDs)	
14.	Flavanone	2,3-dihydroflavanone, 2′-carboxy-5,7-dimethoxy-flavanone, 4′-bromo-5,7-dimethoxy-flavanone	Electron excitation phenomena	Methanol	317	400	2.07×10^5	0.02	great potential use in living cell imaging and mitochondrial imaging ,in vivo imaging of zebrafish,bioimaging	[19]
15.	Styrene	triphenylamine or carbazole-substituted styrene derivatives	conjugations phenomenon	DMSO	490	530	40300	0.037	high thermal stability, opto-electronic properties, charge-transport material.	[20, 21]
16.	Hesperidin	hesperetin, glucosyl hesperidin,	Electron excitation phenomena		284	604	17800	Not detected	in vitro and in vivo based on α-glucosidase inhibition, glucose consumption of HepG2	[22]

									cells, and blood glucose level in streptozotocin-induced diabetic mice	
17.	Herniarin (7-methoxy coumarin) (One of the derivatives of Umbelliferone)		Proton donor acceptor phenomena	DMF	323	398	14,100	0.32	Used in drug delivery systems due to its antioxidant, antiproliferative activities	[23, 24]
18.	Rubrene	5-, 11-phenyls of rubrene,	π stacking with π-conjugated electron system	Benzene	528	620	11800	0.54	Organic light-emitting materials,excellent cell-targeting capability, high stability, and good biocompatibility.	[25, 26]
19.	Matlaline(tetrahydromethanobenzofuro[2,3-*d*]oxacine)		π– π electron transition	H_2O	492	513	5800–33800	1.0	cell biology for the detection of cancer cell	[27, 28]
20.	Scopoletin (6-methoxy-7-hydroxy coumarin)		π–π electronic transiti	H_2O	344	434		0.68	Antioxidant, antidepressant	[29]

			on							
21.	4′,6-diamidino-2-phenylindole (DAPI)	-	Interaction with nucleotides.	H_2O	340	453	2.7×10^{-4}	0.04	DNA-specific probe for flow cytometry, chromosome staining, and, DNA visualization.	[30]
22.	Canthin-6-one	Canthin-5,6-dione, Canthin-2,6-dione	Pie electron excitation	H_2O	434	451	Around 10^{-4}	0.62	less cytotoxic, hence useful cell marker tool.	[31]
23.	Tryptophan (4-methyl tryptophan)	4-azatryptophans,6-cyanotryptophan, 4-cyanotryptophan	protein-protein interations	H_2O	280	341	5600	0.10	optical imaging, novel artifical protein can be design, genetic encolding	[32]
24.	Benzene	1,2,4-Trimethylbenzene,1,3.5-Trimethylbenzene,o-Xylene,	π - π interactions	Cyclohexane	255	210	5000–6200	0.053	organic light-emitting diodes (OLEDS), biological imaging, organic solid-state lasers, chemical sensors	[33]
25.	Beta-carotene	(3R,3′R)-astaxanthin bis(N-Cbz-l-	Electron excitati	Ether + Isopenta	452 nm	539 nm	139500	<0.0001	Reduce the incidenc	[34]

		alanine ester) (3B(and (3S,3′S)-astaxanthin bis(N,N-dimethylglycine ester	on and emission phenomena	ne + Ethanol					e of chronic diseases such as cardiovascular diseases, cancer, cataracts, and neural tube defects	
**26.**	Berberine	13-methylberberine	Excitation phenomena	2-propanol	431	534	5290	0.045	Pharmacological and biochemical activities including cytotoxic activities, anti-parasitic, and anti-inflammatory activities	[35, 36]
**27.**	Hypericin	2,5-dibromohypericin, 2,5,9,12-tetrabromohypericin and perylenequinones,iodohypericin	Photosensitizing phenomena ,donors and acceptors of electrons	DMSO	540	603	3.8×10^8	0.39 – 0.01	potent photosensitizer for photodynamic therapy in both preclinical and clinical settings, necrosis-avid contrast agents	[37]
**28**	cis-parinaric acid	trans-parinaric	π - π * excitata	Ethanol	320	412	47000	0.06 –	characterise	[38]

.		acid	ion					0.22	lipid–protein as well as lipoprotein–protein inter, purifiaction and identification of lipoprotein	
29.	Epicocconone		Charge transfer phenomena	Toluene	444	535	68600	0.074	it is used as protein detection ,Western blotting, proteomic gel staining, protein quantification, live-cell imaging, and monitoring of enzymatic activity	[39]
30.	5’-6 locked nucleosides	1,10-phenanthroline containing nucleoside	Conjugation phenomenon	THF	319	355	8000	0.405	high brightness in fluorescence, used in internal duplex labeling with three strand system	[40]
31	Anthranilates	N-methylanthra	π - π * electro	Ethanol	350	475	4900	0.4–0.65	Chemical	[41,

.		nilate, methyl anthranilate, and methyl N-methylanthranilate	nic transitions u						biology and imaging studies	42]
32.	Oligopyrroles	2-methyl-1,3-di(1H-pyrrol-1-yl) propan-1-one (MDPP)	Electron donor accepter phenomena	DCM	467	564	52200 – 60000	0.089	Oligopyrroles for sensing metal ions and anions , Environmental and biochemical analysis	[43]
33.	Quinone	tetramethyl-1,4-benzoquinone (TMBQ) and 2,6-dimethoxy-1,4-benzoquinone (DMOBQ),1,4-naphthoquinones	Electron excitation phenomena	Benzene, Water, Methyl cyanide	300 – 490	470 – 500	7200	1	cell imaging , biological and chemical field also	[44]
34.	Tyrosine	mono- and bis-styryl-L-tyrosine,3-iodo-L-tyrosie,	Photoexcitation	DMSO	278	352	5300	0.12	Creates optoelectronic devices, examines cellular functions, exploration and anticipation of biological mechanism and function	[45]

**35.**	BODIPY	4,4-Difluoro-4-bora-3a, 4a-diaza-s-indacene	Electron excitations	Toluene	505	515	>50,000	0.19 – 0.22	Tunable electronic and photonic properties,dye-sensitized solar cells (DSSCs)	[46]
**36.**	Cyanine	trimethine cyanine dye (TCy3),		Ethanol	780	830	1.3×10^5	0.01	nucleic acid detection, DNA detection of biological samples, construction of probes	[47]
**37.**	1,8-napthalimide	4-N-methylpiperazine-1,8-napthalimide		Methanol	416	525	3.87×10^{4}	0.013	antimicrobial , antibacterial, and anticancer agents	[48]
**38.**	Pacific blue	6,8-difluoro-7-hydroxy coumarin	excitation and emission	Phenol	400	447	29500	0.75	labelling of proteins	[49]
**39.**	Fluorescein	carboxyfluorescein, fluorescein di-acetate (FDA), carboxyfluorescein di-acetate (CFDA)	Electronic excitations	0.01 M NaOH	490	515	88000	0.93	cellular pH imaging, cancer therapy, bacterial growth, and duel sensor	[50]
**40.**	Phthalocyaninene	Zinc tetra(tert-butylphenoxy)phthalocya	Electronic excitation	DMSO	676	681	1.995×10^{5}	0.20	optical imaging, phototh	[51, 52]

		nine, zinc octachlorophthalocyanine, zinc tetra sulphophthalocyanine							erapy, increase the biodistribution, solubility, stability, and target tissues for biological imaging	
41.	Thioflavin T	Diethyl(phenothiazin-3-ylidene)azanium, Thiopyronine	Absorption and emission phenomenon	H_2O	412	485	36000	0.004–0.028	fibril formation, diagnosis of diseases like Alzheimer's, Parkinson's, type-II diabetes	[53, 54]
42.	Phenosafranin		Electron donor and accepter phenomenon	Methanol	527	567	35600	0.20	therapy and imaging, introducing an antimicrobial film based on cellulose	[55]
43.	Benzimidazolones	5-nitrobenzimidazolone, 5,6,7-trinitrobenzimidazolone	Electronic excitations	Ethanol	286	334	2500	0.34	Used as UV filters in sunscreen, cosmetic formulation.	[56, 57]

44.	Phenanthridine	benzo [4,5]imidazo [1,2-a]thieno[2,3-c]quinoline (BTQ), benzo[4,5]imidazo[1,2-a]furo[2,3-c]quinoline (BFQ), 5,6-dimethylbenzo[4,5]imidazo[1,2-a]furo[2,3-c]quinoline (DFQ)	Electron donor-acceptor phenomenon	DMSO	338	487	35500	0.59	The significant applicable in anticancer and DNA cleavage activity, effective against anticancer cell lines	[58, 59]
45.	Azure II	3,4-Dihydroxyphenylacetic, 2,5-dihydroxyterephtalic		H_2O	657	820	81300	0.04	sed as sensors, biosensors, as mediator for various chemical or biochemical reactions, photosensitizer for microorganism	[60]
46.	4-Chloro-7-nitrobenzofuraza	4-chloro-7-nitrobenzo2-oxa-1,3-diazole, Estrone 3-(7-nitrobenzofurazan) ether	Electron excitation phenomenon on		332 - 450	524 – 645	8130	Not detected	mainly used for cell imaging and biomedical application	
47.	Merocyanine 540	butyl Merocyanine UO,the sulfonate group replaced with a	Photoisomerization process	$CDCl_3$	559	600	138000	0.39	inhibit glial cell aggregation, thereby reducing the	[61, 62]

		methyl group							formation of glial cells its main use	
48.	Pyrene	1,3,6,8-tetrakis(4-n-monoethyleneglycol-(2-propynyl)ether)pyrene , 1,3,6,8-tetrakis(4-n-diethyleneglycol-(2-propynyl)ether)pyrene and	dipolar donor-π-acceptor (D-π-A)	Methanol	378	422	100000	0.99	Strong emission of fluorescence in live cells, detection of reactive oxygen species (ROS)	[82]
49.	Isophthalamide	2-{4-[(1H-1,2,4-triazol-1-yl)methyl]phenyl}-1H–isoindole-1,3(2H)-dione			415	510			used to increase the drug resistance, anticancer drug	[63]
50.	Phenothiazine	6,7-diphenyl-[1,2,5] thiadiazolo[3,4-g] quinoxaline -ph phenothiazine (DPTQ-phPTZ)	Electron donor accepter phenomena	Cyclohexane	350 – 850	911	0.27×10^{-4}	0.148 (Observed in non-polar cyclohexane)	decreases the growth and survival of aggressive cancer cells (glioblastoma) by inducing the breakdown of double DNA strand, leads to apoptosis and autopha	[64]

									gic cell death, in addition to G1 phase of cell cycle arrest.	
51.	Rhodamine	Si-rhodamine, 4-carboxyrhodamines	donor-acceptor of electron	PBS	720	757	1.069 $\times10^5$	0.8-0.92	labeling of intracellular structures, isomeric tuning Halo-tagged and SNAP-tagged proteins	[65]
52.	Piroxicam	4-hydroxy-2-methyll-2H-1,2-benzathinezine-1-(N-(2-pyridinyl)carboxamide)-1,1-dioxide	Electron excitation phenomenaa	Methanol	326	478	13000	0.035	It is used for the determination of cancer cell and other living organism diseases	[66]
53.	Photofrin	Haematoporphyrin derivative	pi-pi stacking interaction	Toluene	400	693	8535	<0.01	treatment of solid tumors, treatment of esophageal, lung, pancreatic, brain, skin, breast, and bladder	[67]

									cancer	
54.	1,8-napthalimide	4-N-methylpiperazine-1,8-napthalimide		Methanol	416	525	3.87 $\times 10^4$	0.013	antimicrobial, antibacterial, and anticancer agents	[48]
55.	p-Aminophenol	Fluorofluorophores (Chromene, Squaraine, Oxazine)	Electronic excitation	EtOH	641	664	1.68$\times 10^5$	0.03	Allows the creation of extremely fluorescent nanoemulsions; holds promise as brilliant bio-imaging agents	[68]
56.	Rhodamine 123	4,5-dibromorhodamine methyl ester (dye 2) and 4,5-dibromorhodamine n -butyl ester (dye 3)	Electron excitation phenomenon	Methanol	507	600	82100	0.86	It is used for the detection of live cell imaging, mitochondria analysis	[69]
57.	Squarylium dye III	indodicarbocyanine dye D3, squarylium dye D1	Donar - acceptor - Donar of electron	Methylene chloride	627.6	676	30900	0.65	photosensitizers, and biolabeling and chemosensory materials for analytical uses in biomedi	[70]

									cal field	
58.	1,2,4-trioxolane	4H-1,2,4-triazole,4-alkyl-3,5-bis(4-bromophenyl)-4H-1,2,4-triazoles	π-conjugated systems	CH_2Cl_2	472	545	5×10^5	>0.98	Used for the detection of cell morphological changes, anticancer agent, synthesis of more derivatives	[71, 72]
59.	Hemicyanine		donor-π-acceptor	Ethyl acetate	498	559.2	15,500	0.89	Superoxide Anion Detection, bioimaging and biomedicine	[73, 74]
60.	Alizarin	alizarin-3-methylimino diacetic acid,1,4-dihydroxyanthraquinone and 1,8-dihydroxyanthraquinon	excitation phenomena	DMSO: H_2O	421<br>499	561<br>643	4900<br>16500	0.002<br>0.06	removal of organic pollutants and heavy metal ions from wastewater, reducing bacterial infection in biomedical applications,	[75, 76]

S.N. denotes the number of structures listed.

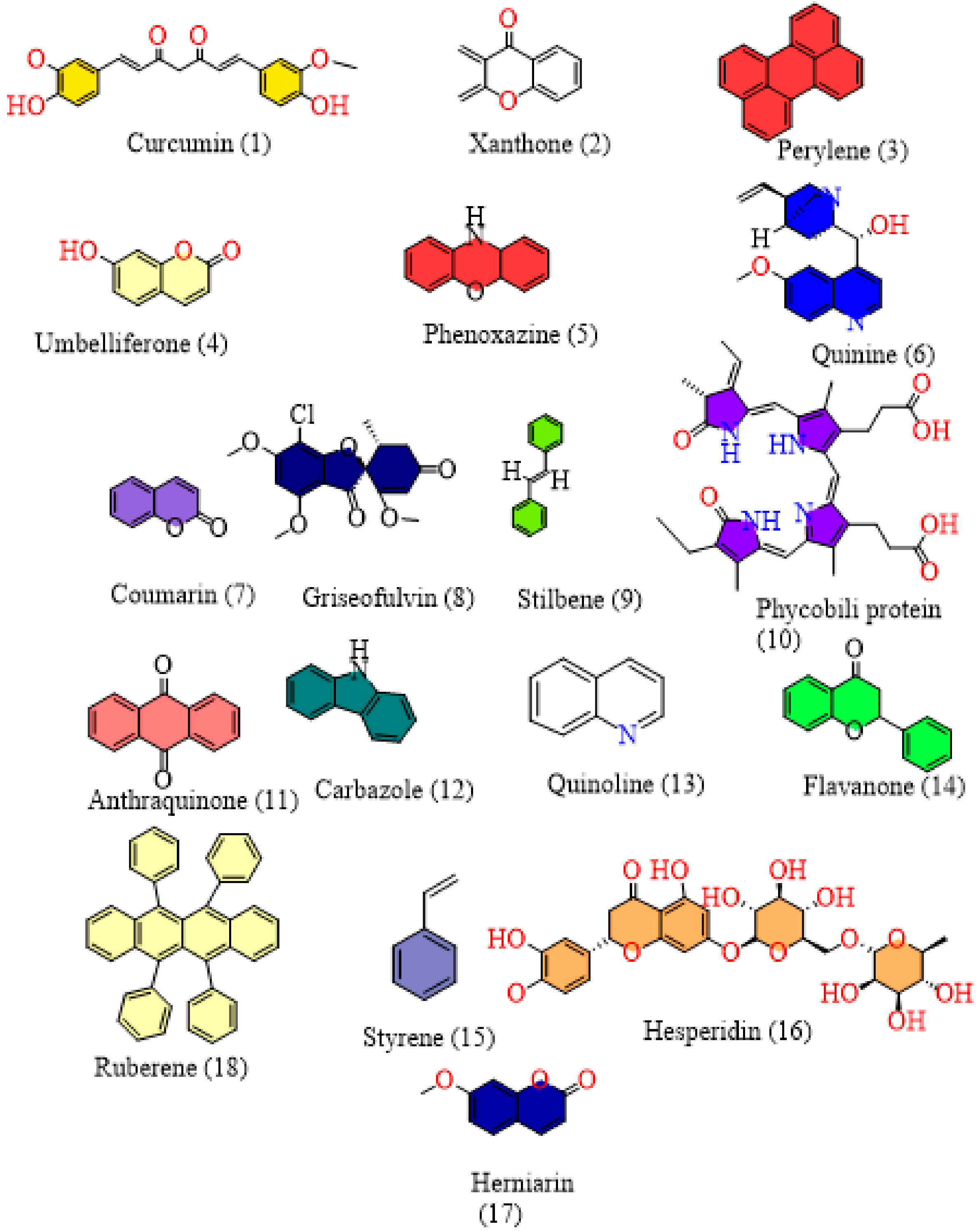
Curcumin (1)
Xanthone (2)
Perylene (3)
Umbelliferone (4)
Phenoxazine (5)
Quinine (6)
Coumarin (7)
Griseofulvin (8)
Stilbene (9)
Phycobili protein (10)
Anthraquinone (11)
Carbazole (12)
Quinoline (13)
Flavanone (14)
Ruberene (18)
Styrene (15)
Hesperidin (16)
Herniarin (17)

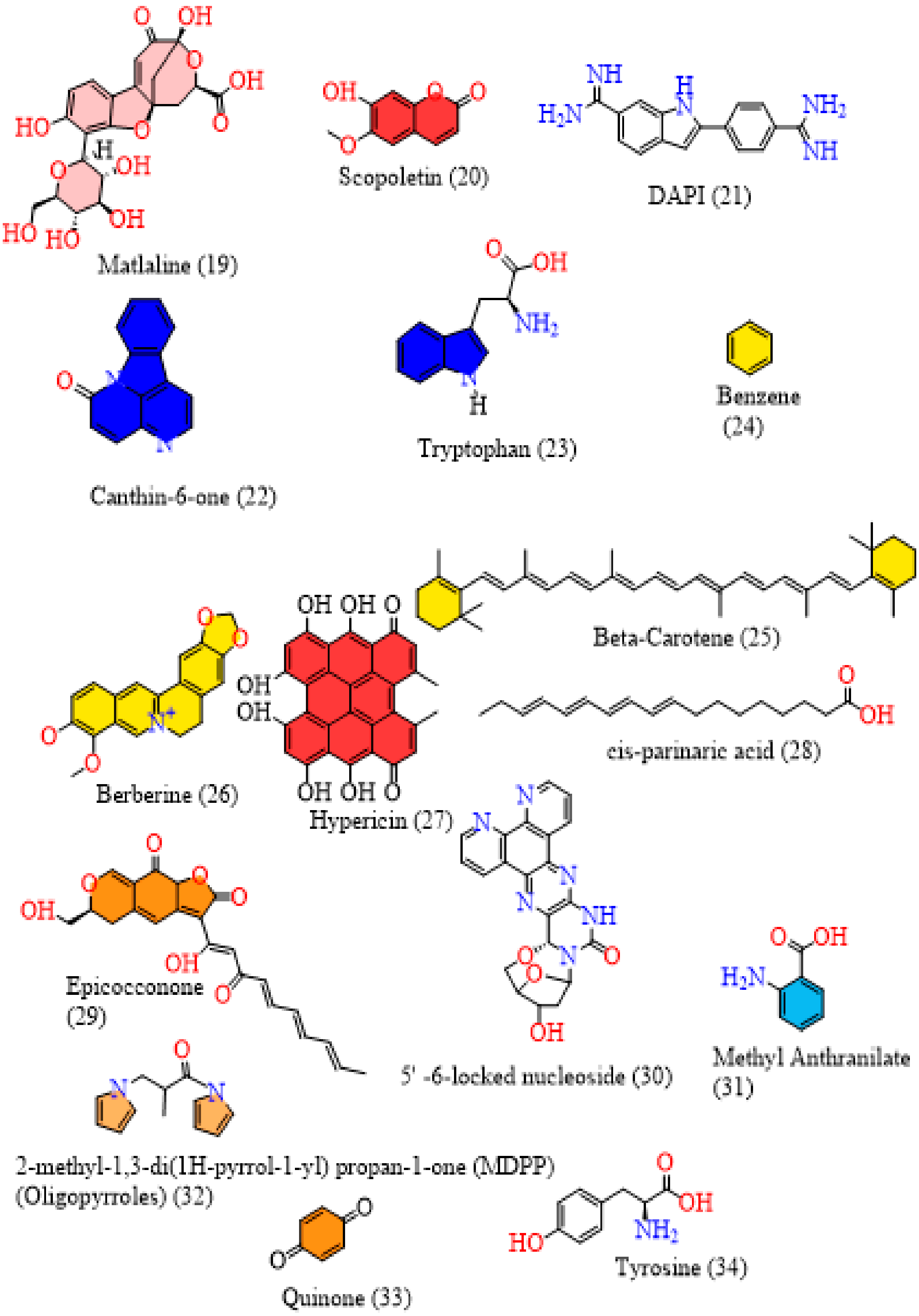

Figure S1: Structures of the listed natural fluorescent compounds

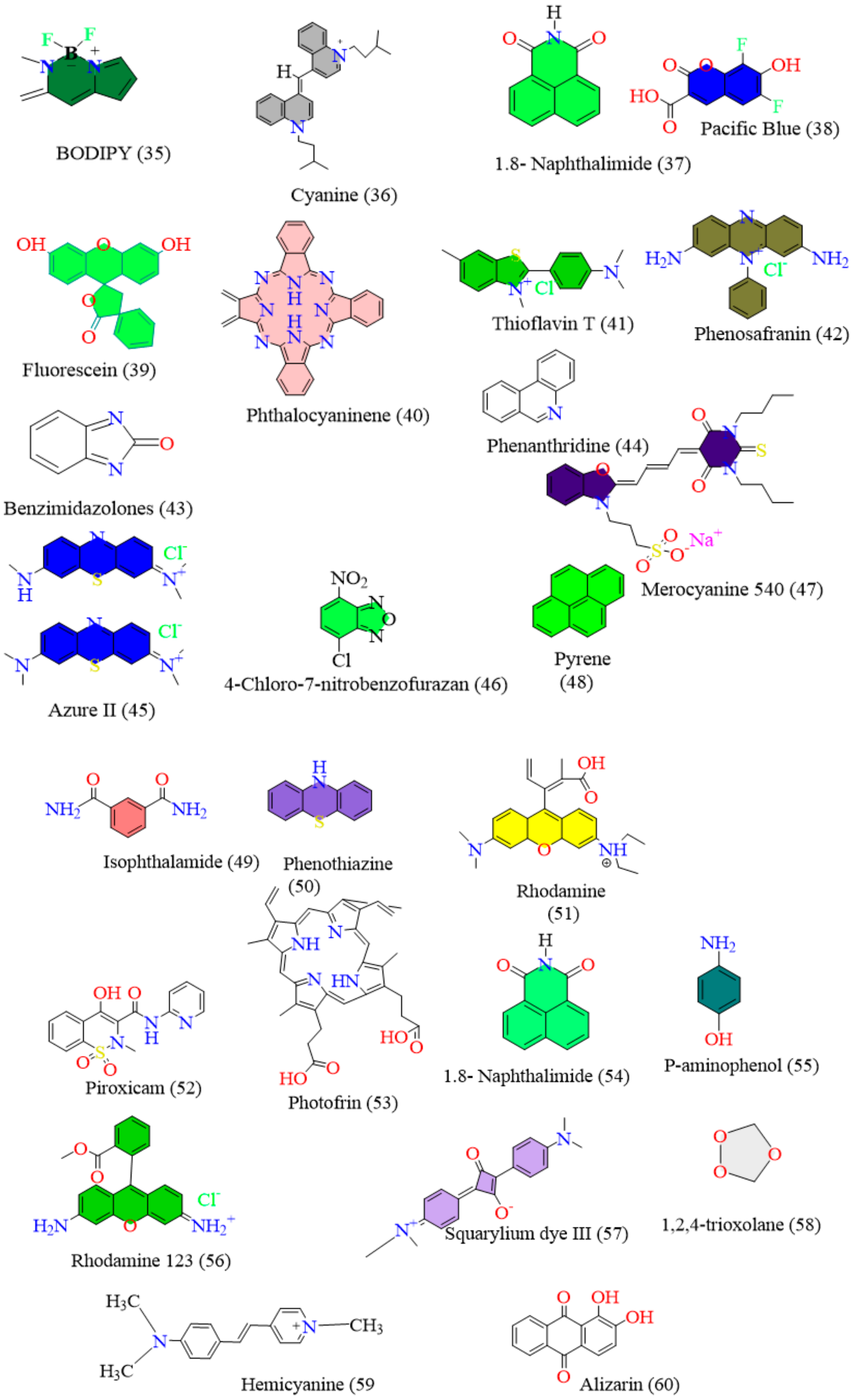

Figure S2: Structures of the listed synthetic fluorescent compounds

References:

1. Jun, J.V.; Chenoweth, D.M.; Petersson, E.J. Rational Design of Small Molecule Fluorescent Probes for Biological Applications. *Org. Biomol. Chem.* **2020**, *18*, 5747–5763, doi:10.1039/D0OB01131B.
2. Ley, C. Photochemical Processes of Superbase Generation in Xanthone Carboxylic Salts - Ley - 2023 - Angewandte Chemie - Wiley Online Library Available online: https://onlinelibrary.wiley.com/doi/10.1002/ange.202214784 (accessed on 2 May 2024).
3. Pinto, M.M.M.; Sousa, M.E.; Nascimento, M.S.J. Xanthone Derivatives: New Insights in Biological Activities. *Current Medicinal Chemistry 12*, 2517–2538.
4. Rostami-Tapeh-Esmail, E.; Golshan, M.; Salami-Kalajahi, M.; Roghani-Mamaqani, H. Perylene-3,4,9,10-Tetracarboxylic Diimide and Its Derivatives: Synthesis, Properties and Bioapplications. *Dyes and Pigments* **2020**, *180*, 108488, doi:10.1016/j.dyepig.2020.108488.
5. Kornicka, A.; Balewski, Ł.; Lahutta, M.; Kokoszka, J. Umbelliferone and Its Synthetic Derivatives as Suitable Molecules for the Development of Agents with Biological Activities: A Review of Their Pharmacological and Therapeutic Potential. *Pharmaceuticals* **2023**, *16*, 1732, doi:10.3390/ph16121732.
6. Gao, M.; Yu, F.; Lv, C.; Choo, J.; Chen, L. Fluorescent Chemical Probes for Accurate Tumor Diagnosis and Targeting Therapy. *Chem. Soc. Rev.* **2017**, *46*, 2237–2271, doi:10.1039/C6CS00908E.
7. Li, S.; Cheng, T.; Yin, C.; Zhou, S.; Fan, Q.; Wu, W.; Jiang, X. Phenothiazine versus Phenoxazine: Structural Effects on the Photophysical Properties of NIR-II AIE Fluorophores. *ACS Appl. Mater. Interfaces* **2020**, *12*, 43466–43473, doi:10.1021/acsami.0c12773.
8. Instrumentation for Fluorescence Spectroscopy. In *Principles of Fluorescence Spectroscopy*; Lakowicz, J.R., Ed.; Springer US: Boston, MA, 2006; pp. 27–61 ISBN 978-0-387-46312-4.
9. Petersen, A.B.; Andersen, N.S.; Konotop, G.; Hanafiah, N.H.M.; Raab, M.S.; Krämer, A.; Clausen, M.H. Synthesis and Formulation Studies of Griseofulvin Analogues with Improved Solubility and Metabolic Stability. *European Journal of Medicinal Chemistry* **2017**, *130*, 240–247, doi:10.1016/j.ejmech.2017.02.055.
10. Townley, E.R. Griseofulvin. In *Analytical Profiles of Drug Substances*; Florey, K., Ed.; Academic Press, 1979; Vol. 8, pp. 219–249.
11. Mahmoodi, A.; Panahi, F.; Eshghi, F.; Kimiaei, E. A Novel Tetra-Stilbene-Based Fluorescent Compound: Synthesis, Characterization and Photophysical Properties Evaluation. *Journal of Luminescence* **2018**, *199*, 165–173, doi:10.1016/j.jlumin.2018.03.033.
12. Scheer, H.; Yang, X.; Zhao, K.-H. Biliproteins and Their Applications in Bioimaging. *Procedia Chemistry* **2015**, *14*, 176–185, doi:10.1016/j.proche.2015.03.026.

13. Vinothkanna, A.; Sekar, S. Diagnostic Applications of Phycobiliproteins. In *Pigments from Microalgae Handbook*; Jacob-Lopes, E., Queiroz, M.I., Zepka, L.Q., Eds.; Springer International Publishing: Cham, 2020; pp. 585–610 ISBN 978-3-030-50971-2.
14. Wu, M.; Goodwin, P.M.; Ambrose, W.P.; Keller, R.A. Photochemistry and Fluorescence Emission Dynamics of Single Molecules in Solution: B-Phycoerythrin. *J. Phys. Chem.* **1996**, *100*, 17406–17409, doi:10.1021/jp9616775.
15. Singh, A.J.; Gorka, A.P.; Bokesch, H.R.; Wamiru, A.; O'Keefe, B.R.; Schnermann, M.J.; Gustafson, K.R. Harnessing Natural Product Diversity for Fluorophore Discovery: Naturally Occurring Fluorescent Hydroxyanthraquinones from the Marine Crinoid Pterometra Venusta. *J. Nat. Prod.* **2018**, *81*, 2750–2755, doi:10.1021/acs.jnatprod.8b00761.
16. Yin, J.; Ma, Y.; Li, G.; Peng, M.; Lin, W. A Versatile Small-Molecule Fluorescence Scaffold: Carbazole Derivatives for Bioimaging. *Coordination Chemistry Reviews* **2020**, *412*, 213257, doi:10.1016/j.ccr.2020.213257.
17. Zhang, Q.; Zhu, W.; Fang, M.; Yin, F.; Li, C. Synthesis, Photophysical and Electrochemical Properties of Two Novel Carbazole-Based Dye Molecules. *Spectrochimica Acta Part A: Molecular and Biomolecular Spectroscopy* **2015**, *135*, 379–385, doi:10.1016/j.saa.2014.06.159.
18. Lewinksa Application of Quinoline Derivatives in Third-Generation Photovoltaics | Journal of Materials Science: Materials in Electronics Available online: https://link.springer.com/article/10.1007/s10854-021-06225-6 (accessed on 2 May 2024).
19. Li, N.; Liu, L.; Luo, H.; Wang, H.; Yang, D.; He, F. Flavanone-Based Fluorophores with Aggregation-Induced Emission Enhancement Characteristics for Mitochondria-Imaging and Zebrafish-Imaging. *Molecules* **2020**, *25*, 3298, doi:10.3390/molecules25143298.
20. Kim, M.J.; Li, Y.; Junge, J.A.; Kim, N.K.; Fraser, S.E.; Zhang, C. Development of Highly Fluorogenic Styrene Probes for Visualizing RNA in Live Cells. *ACS Chem. Biol.* **2023**, *18*, 1523–1533, doi:10.1021/acschembio.3c00141.
21. Mu, X.; Han, L.; Leng, X.; Li, Y. Synthesis of Functional Polyethylene via Scandium Catalysted Copolymerization of Ethylene with Triphenylamine- or Carbazole-Substituted Styrene Derivatives. *Polymer* **2022**, *241*, 124548, doi:10.1016/j.polymer.2022.124548.
22. Taniguchi, M.; LaRocca, C.A.; Bernat, J.D.; Lindsey, J.S. Digital Database of Absorption Spectra of Diverse Flavonoids Enables Structural Comparisons and Quantitative Evaluations. *J. Nat. Prod.* **2023**, *86*, 1087–1119, doi:10.1021/acs.jnatprod.2c00720.
23. Jasim, L.M.M.; Tabrizi, M.H.; Darabi, E.; Jaseem, M.M.M. The Antioxidant, Anti-Angiogenic, and Anticancer Impact of Chitosan-Coated Herniarin-Graphene Oxide Nanoparticles (CHG-NPs). *Heliyon* **2023**, *9*, doi:10.1016/j.heliyon.2023.e20042.

24. Nizomov, N.; Kholov, A.U.; Ishchenko, A.A.; Ishchenko, V.V.; Khilya, V.P. Electronic Structure and Spectral Fluorescence Properties of Umbelliferone and Herniarin. *J Appl Spectrosc* **2007**, *74*, 626–634, doi:10.1007/s10812-007-0102-z.

25. Ji, L.-F.; Fan, J.-X.; Qin, G.-Y.; Zhang, N.-X.; Lin, P.-P.; Ren, A.-M. Theoretical Study on the Electronic Structures and Charge Transport Properties of a Series of Rubrene Derivatives. *J. Phys. Chem. C* **2018**, *122*, 21226–21238, doi:10.1021/acs.jpcc.8b07018.

26. Löhmannsröben, H.-G. Photophysical Properties and Laser Performance of Rubrene. *Appl. Phys. B* **1988**, *47*, 195–199, doi:10.1007/BF00684088.

27. Acuña, A.U.; Amat-Guerri, F.; Morcillo, P.; Liras, M.; Rodríguez, B. Structure and Formation of the Fluorescent Compound of Lignum Nephriticum. *Org. Lett.* **2009**, *11*, 3020–3023, doi:10.1021/ol901022g.

28. Hernandez-Martinez, Á.R.; Molina, G.A.; Rodríguez-Torres, A.; Ledesma-Mendoza, B.; Del Real, A.; Barroso-Flores, J.; Estevez, M. Fluorescence Decay Rate of Selected Compounds from *Eysenhardtia Polystachya* Extracts and Their Viability as Biosensors. *Materials Science and Engineering: C* **2019**, *104*, 109978, doi:10.1016/j.msec.2019.109978.

29. Pham, H.T.; Yoo, J.; VandenBerg, M.; Muyskens, M.A. Fluorescence of Scopoletin Including Its Photoacidity and Large Stokes Shift. *J Fluoresc* **2020**, *30*, 71–80, doi:10.1007/s10895-019-02471-4.

30. Kapuscinski, J. DAPI: A DNA-Specific Fluorescent Probe. *Biotechnic & Histochemistry* **1995**, *70*, 220–233, doi:10.3109/10520299509108199.

31. dos Santos Costa, R.; do Espírito-Santo, R.F.; Abreu, L.S.; de Oliveira Aguiar, L.; Leite Fontes, D.; Fechine Tavares, J.; Sobral da Silva, M.; Botelho Pereira Soares, M.; da Silva Velozo, E.; Flora Villarreal, C. Fluorescent Canthin-6-One Alkaloids from Simaba Bahiensis: Isolation, Identification, and Cell-Labeling Properties. *ChemPlusChem* **2019**, *84*, 260–267, doi:10.1002/cplu.201800591.

32. Acharyya, A.; Zhang, W.; Gai, F. Tryptophan as a Template for Development of Visible Fluorescent Amino Acids. *J. Phys. Chem. B* **2021**, *125*, 5458–5465, doi:10.1021/acs.jpcb.1c02321.

33. Quina, F.H.; Carroll, F.A. Radiative and Nonradiative Transitions in Solution. First Excited Singlet State of Benzene and Its Methyl Derivatives. *J. Am. Chem. Soc.* **1976**, *98*, 6–9, doi:10.1021/ja00417a002.

34. Cherry, R.J.; Chapman, D.; Langelaar, J. Fluorescence and Phosphorescence of β-Carotene. *Trans. Faraday Soc.* **1968**, *64*, 2304–2307, doi:10.1039/TF9686402304.

35. Díaz, M.S.; Freile, M.L.; Gutiérrez, M.I. Solvent Effect on the UV/Vis Absorption and Fluorescence Spectroscopic Properties of Berberine. *Photochem Photobiol Sci* **2009**, *8*, 970–974, doi:10.1039/b822363g.

36. Mikeš, V.; Dadák, V. Berberine Derivatives as Cationic Fluorescent Probes for the Investigation of the Energized State of Mitochondria. *Biochimica et Biophysica Acta (BBA) - Bioenergetics* **1983**, *723*, 231–239, doi:10.1016/0005-2728(83)90122-6.
37. Jiang, B.; Wang, J.; Ni, Y.; Chen, F. Necrosis Avidity: A Newly Discovered Feature of Hypericin and Its Preclinical Applications in Necrosis Imaging. *Theranostics* **2013**, *3*, 667–676, doi:10.7150/thno.6650.
38. Narayanaswami, V.; McNamee, M.G. Protein-Lipid Interactions and Torpedo Californica Nicotinic Acetylcholine Receptor Function. 2. Membrane Fluidity and Ligand-Mediated Alteration in the Accessibility of .Gamma. Subunit Cysteine Residues to Cholesterol. *Biochemistry* **1993**, *32*, 12420–12427, doi:10.1021/bi00097a021.
39. Peixoto, P.A.; Boulangé, A.; Ball, M.; Naudin, B.; Alle, T.; Cosette, P.; Karuso, P.; Franck, X. Design and Synthesis of Epicocconone Analogues with Improved Fluorescence Properties. *J. Am. Chem. Soc.* **2014**, *136*, 15248–15256, doi:10.1021/ja506914p.
40. Gislason, K.; Gophane, D.B.; Sigurdsson, S.T. Syntheses and Photophysical Properties of 5′–6-Locked Fluorescent Nucleosides. *Org. Biomol. Chem.* **2012**, *11*, 149–157, doi:10.1039/C2OB26536B.
41. Duval, R.; Duplais, C. Fluorescent Natural Products as Probes and Tracers in Biology. *Nat. Prod. Rep.* **2017**, *34*, 161–193, doi:10.1039/C6NP00111D.
42. Lee, H.L.; Kim, S.-Y.; Kim, E.J.; Han, D.Y.; Kim, B.-G.; Ahn, J.-H. Synthesis of Methylated Anthranilate Derivatives Using Engineered Strains of Escherichia Coli. **2019**, *29*, 839–844, doi:10.4014/jmb.1904.04022.
43. Zhang, S.; Lv, G.; Wang, G.; Zhu, K.; Yu, D.; Shao, J.; Wang, Y.; Liu, Y. Facile Preparation and Fluorescence Properties of a Soluble Oligopyrrole Derivative. *Journal of Photochemistry and Photobiology A: Chemistry* **2015**, *309*, 30–36, doi:10.1016/j.jphotochem.2015.04.023.
44. Barbafina, A.; Elisei, F.; Latterini, L.; Milano, F.; Agostiano, A.; Trotta, M. Photophysical Properties of Quinones and Their Interaction with the Photosynthetic Reaction Centre. *Photochem Photobiol Sci* **2008**, *7*, 973–978, doi:10.1039/b805897k.
45. Cheruku, P.; Huang, J.-H.; Yen, H.-J.; Iyer, R.S.; Rector, K.D.; Martinez, J.S.; Wang, H.-L. Tyrosine-Derived Stimuli Responsive, Fluorescent Amino Acids. *Chem. Sci.* **2015**, *6*, 1150–1158, doi:10.1039/C4SC02753A.
46. Poddar, M.; Misra, R. Recent Advances of BODIPY Based Derivatives for Optoelectronic Applications. *Coordination Chemistry Reviews* **2020**, *421*, 213462, doi:10.1016/j.ccr.2020.213462.

47. Ma, X.; Shi, L.; Zhang, B.; Zhao, S.; Yuan, X.; Zhang, X. Cy3 Cyanine Dye with Strong Fluorescence Enhancement for AGRO100 and Its Derivative. *J. Phys. Chem. B* **2023**, *127*, 1811–1818, doi:10.1021/acs.jpcb.2c08784.
48. Cangiotti, M.; Staneva, D.; Ottaviani, M.F.; Vasileva-Tonkova, E.; Grabchev, I. Synthesis and Characterization of Fluorescent PAMAM Dendrimer Modified with 1,8-Naphthalimide Units and Its Cu(II) Complex Designed for Specific Biomedical Application. *Journal of Photochemistry and Photobiology A: Chemistry* **2021**, *415*, 113312, doi:10.1016/j.jphotochem.2021.113312.
49. Lee, M.M.; Peterson, B.R. Quantification of Small Molecule–Protein Interactions Using FRET between Tryptophan and the Pacific Blue Fluorophore. *ACS Omega* **2016**, *1*, 1266–1276, doi:10.1021/acsomega.6b00356.
50. Le Guern, F.; Mussard, V.; Gaucher, A.; Rottman, M.; Prim, D. Fluorescein Derivatives as Fluorescent Probes for pH Monitoring along Recent Biological Applications. *International Journal of Molecular Sciences* **2020**, *21*, 9217, doi:10.3390/ijms21239217.
51. de Souza, T.F.M.; Torres Antonio, F.C.; Homem-de-Mello, P.; Ribeiro, A.O. Unsymmetrical Zinc (II) Phthalocyanine and Zinc (II) Naphthalocyanine with 2,3-Dicyano-1,4-Diphenylnaphthalene Precursor. *Dyes and Pigments* **2020**, *172*, 107824, doi:10.1016/j.dyepig.2019.107824.
52. Zhang, Y.; Lovell, J.F. Recent Applications of Phthalocyanines and Naphthalocyanines for Imaging and Therapy. *WIREs Nanomedicine and Nanobiotechnology* **2017**, *9*, e1420, doi:10.1002/wnan.1420.
53. Hsu, J.C.-C.; Chen, E.H.-L.; Snoeberger, R.C.I.; Luh, F.Y.; Lim, T.-S.; Hsu, C.-P.; Chen, R.P.-Y. Thioflavin T and Its Photoirradiative Derivatives: Exploring Their Spectroscopic Properties in the Absence and Presence of Amyloid Fibrils. *J. Phys. Chem. B* **2013**, *117*, 3459–3468, doi:10.1021/jp309331u.
54. Mukherjee, P.; Rafiq, S.; Sen, P. Dual Relaxation Channel in Thioflavin-T: An Ultrafast Spectroscopic Study. *Journal of Photochemistry and Photobiology A: Chemistry* **2016**, *328*, 136–147, doi:10.1016/j.jphotochem.2016.05.012.
55. Porcal, G.V.; Arbeloa, E.M.; Orallo, D.E.; Bertolotti, S.G.; Previtali, C.M. Photophysics of Safranine-O and Phenosafranine in Reverse Micelles of BHDC. *Journal of Photochemistry and Photobiology A: Chemistry* **2011**, *226*, 51–56, doi:10.1016/j.jphotochem.2011.10.014.
56. Lazar, Z.; Benali, B.; Elblidi, K.; Zenkouar, M.; Lakhrissi, B.; Massoui, M.; Kabouchi, B.; Cazeau-Dubroca, C. Photophysical Study of Benzimidazolone and Its Derivative Molecules in Solution. *Journal of Molecular Liquids* **2003**, *106*, 89–95, doi:10.1016/S0167-7322(03)00023-0.
57. V., M.; Kulkarni, M.V.; Badami, S.; Yenagi, J.; Tonannavar, J. Effect of Nitro Groups on the Photo Physical Properties of Benzimidazolone: A Solvatochromic Study. *Spectrochimica Acta Part A: Molecular and Biomolecular Spectroscopy* **2011**, *84*, 137–143, doi:10.1016/j.saa.2011.09.021.

58. Liu, J.; Yue, Y.; Wang, J.; Yan, X.; Liu, R.; Sun, Y.; Li, X. Study of Interaction between Human Serum Albumin and Three Phenanthridine Derivatives: Fluorescence Spectroscopy and Computational Approach. *Spectrochimica Acta Part A: Molecular and Biomolecular Spectroscopy* **2015**, *145*, 473–481, doi:10.1016/j.saa.2015.03.069.
59. Sawminathan, S.; Munusamy, S.; Jothi, D.; Iyer, S.K. Phenanthridine-Based Donor/Acceptor Fluorescent Dyes: Synthesis, Photophysical Properties and Fluorometric Sensing of Biogenic Primary Amines. *ChemistrySelect* **2021**, *6*, 858–864, doi:10.1002/slct.202004040.
60. Paul, P.; Suresh Kumar, G. Spectroscopic Studies on the Binding Interaction of Phenothiazinium Dyes Toluidine Blue O, Azure A and Azure B to DNA. *Spectrochimica Acta Part A: Molecular and Biomolecular Spectroscopy* **2013**, *107*, 303–310, doi:10.1016/j.saa.2013.01.063.
61. Benniston Photophysical Properties of Merocyanine 540 Derivatives - Journal of the Chemical Society, Faraday Transactions (RSC Publishing) Available online: https://pubs.rsc.org/en/content/articlelanding/1994/ft/ft9949000953 (accessed on 2 May 2024).
62. Williamson, P.; Mattocks, K.; Schlegel, R.A. Merocyanine 540, a Fluorescent Probe Sensitive to Lipid Packing. *Biochimica et Biophysica Acta (BBA) - Biomembranes* **1983**, *732*, 387–393, doi:10.1016/0005-2736(83)90055-X.
63. Malla, J.A.; Umesh, R.M.; Yousf, S.; Mane, S.; Sharma, S.; Lahiri, M.; Talukdar, P. A Glutathione Activatable Ion Channel Induces Apoptosis in Cancer Cells by Depleting Intracellular Glutathione Levels. *Angewandte Chemie International Edition* **2020**, *59*, 7944–7952, doi:10.1002/anie.202000961.
64. Omoruyi, S.I.; Ekpo, O.E.; Semenya, D.M.; Jardine, A.; Prince, S. Exploitation of a Novel Phenothiazine Derivative for Its Anti-Cancer Activities in Malignant Glioblastoma. *Apoptosis* **2020**, *25*, 261–274, doi:10.1007/s10495-020-01594-5.
65. Bucevičius, J.; Gerasimaitė, R.; Kiszka, K.A.; Pradhan, S.; Kostiuk, G.; Koenen, T.; Lukinavičius, G. A General Highly Efficient Synthesis of Biocompatible Rhodamine Dyes and Probes for Live-Cell Multicolor Nanoscopy. *Nat Commun* **2023**, *14*, 1306, doi:10.1038/s41467-023-36913-2.
66. Al-Kindy, S.M.Z.; Al-Wishahi, A.; Suliman, F.E.O. A Sequential Injection Method for the Determination of Piroxicam in Pharmaceutical Formulations Using Europium Sensitized Fluorescence. *Talanta* **2004**, *64*, 1343–1350, doi:10.1016/j.talanta.2004.04.014.
67. Myrzakhmetov, B.; Arnoux, P.; Mordon, S.; Acherar, S.; Tsoy, I.; Frochot, C. Photophysical Properties of Protoporphyrin IX, Pyropheophorbide-a, and Photofrin® in Different Conditions. *Pharmaceuticals* **2021**, *14*, 138, doi:10.3390/ph14020138.

68. M Ellen Fluorofluorophores: Fluorescent Fluorous Chemical Tools Spanning the Visible Spectrum | Journal of the American Chemical Society Available online: https://pubs.acs.org/doi/10.1021/ja507848f (accessed on 2 May 2024).
69. Pal, P.; Zeng, H.; Durocher, G.; Girard, D.; Li, T.; Gupta, A.K.; Giasson, R.; Blanchard, L.; Gaboury, L.; Balassy, A.; et al. Phototoxicity of Some Bromine-Substituted Rhodamine Dyes: Synthesis, Photophysical Properties and Application as Photosensitizers. *Photochem & Photobiology* **1996**, *63*, 161–168, doi:10.1111/j.1751-1097.1996.tb03008.x.
70. Law, K.Yee. Squaraine Chemistry: Effects of Structural Changes on the Absorption and Multiple Fluorescence Emission of Bis[4-(Dimethylamino)Phenyl]Squaraine and Its Derivatives. *J. Phys. Chem.* **1987**, *91*, 5184–5193, doi:10.1021/j100304a012.
71. Olesiejuk, M.; Kudelko, A.; Świątkowski, M. Highly Luminescent 4H-1,2,4-Triazole Derivatives: Synthesis, Molecular Structure and Photophysical Properties. *Materials* **2020**, *13*, 5627, doi:10.3390/ma13245627.
72. Prodea, A.; Milan, A.; Mioc, M.; Mioc, A.; Oprean, C.; Racoviceanu, R.; Negrea-Ghiulai, R.; Mardale, G.; Avram, Ştefana; Balan-Porcăraşu, M.; et al. Novel Betulin-1,2,4-Triazole Derivatives Promote In Vitro Dose-Dependent Anticancer Cytotoxicity. *Processes* **2024**, *12*, 24, doi:10.3390/pr12010024.
73. Hanazawa, M.; Sumiya, R.; Horikawa, Y.; Irie, M. Thermally Irreversible Photochromic Systems. Reversible Photocyclization of 1,2-Bis (2-Methylbenzo[b]Thiophen-3-Yl)Perfluorocyclocoalkene Derivatives. *J. Chem. Soc., Chem. Commun.* **1992**, 206–207, doi:10.1039/C39920000206.
74. Jędrzejewska, B.; Kabatc, J.; Pietrzak, M.; Pączkowski, J. Hemicyanine Dyes: Synthesis, Structure and Photophysical Properties. *Dyes and Pigments* **2003**, *58*, 47–58, doi:10.1016/S0143-7208(03)00035-4.
75. Anoua, R.; Touhtouh, S.; Rkhis, M.; El Jouad, M.; Hajjaji, A.; Belhora, F.; Bakasse, M.; Sahraoui, B.; Płóciennik, P.; Zawadzka, A. Optical and Electronic Properties of the Natural *Alizarin* Dye: Theoretical and Experimental Investigations for DSSCs Application. *Optical Materials* **2022**, *127*, 112113, doi:10.1016/j.optmat.2022.112113.
76. Grazia, C.; Clementi, C.; Miliani, C.; Romani, A. Photophysical Properties of Alizarin and Purpurin Al(III) Complexes in Solution and in Solid State. *Photochem Photobiol Sci* **2011**, *10*, 1249–1254, doi:10.1039/c1pp05039g.
